# Physiological pH Transition‐Driven Protein Corona Dynamics Regulate Cellular Uptake and Inflammatory Responses of Silica Nanoparticles

**DOI:** 10.1002/advs.202502788

**Published:** 2025-09-03

**Authors:** Yuting Ge, Fangqin Fu, Yu Gao, Tianchang He, Volker Mailänder, Daniel Crespy, Katharina Landfester, Shuai Jiang

**Affiliations:** ^1^ Key Laboratory of Marine Drugs, Chinese Ministry of Education, School of Medicine and Pharmacy Ocean University of China Qingdao 266003 P. R. China; ^2^ Laboratory for Marine Drugs and Bioproducts Qingdao Marine Science and Technology Center Qingdao 266237 P. R. China; ^3^ Max Planck Institute for Polymer Research Ackermannweg 10 55128 Mainz Germany; ^4^ Department of Materials Science and Engineering School of Molecular Science and Engineering Vidyasirimedhi Institute of Science and Technology (VISTEC) Rayong 21210 Thailand

**Keywords:** anti‐tumor therapy, nanomedicine, protein corona, targeting, tumor microenvironment

## Abstract

Protein corona alters the biological identities and interactions of nanoparticles with cells, needing to be thoroughly scrutinized before in vivo applications. Importantly, protein corona is evolving as nanoparticles cross different microenvironments, leading to unpredictable biological behaviors. Unveiling how physiological conditions change, especially pH changes associated with tumor‐targeted delivery, affect protein corona composition and subsequent bio‐interactions, is thus essential for understanding the bio‐fate and therapeutic efficacy of nanomedicines. This study investigates how physiological pH transitions influence protein corona dynamics on silica nanoparticles, cellular uptake, and inflammatory responses. Incubating nanoparticle‐protein corona complexes at different pH values reveals that acidic pH increases protein adsorption and induces structural changes of adsorbed proteins, enhancing uptake by macrophages (RAW264.7 and dTHP‐1) and tumor cells (A549) due to reduced electrostatic repulsion and enhanced membrane interactions. Despite increased uptake at acidic pHs, inflammatory responses of dTHP‐1 cells are reduced as indicated by lower levels of reactive oxygen species and pro‐inflammatory cytokines (e.g., IL‐1*β*, TNF‐*α*, and IL‐6). This is consistent with altered protein corona composition, featuring decreased levels of complement protein C3 and immunoglobulins, and increased regulatory proteins (e.g., C4BPA). These findings highlight the crucial role of microenvironmental pH in modulating protein corona fingerprints and in vivo behaviors of nanomedicines.

## Graphical abstract

Physiological pH transitions modulate protein corona dynamics on nanoparticles, altering their cellular uptake and inflammatory responses. Acidic pHs enhance protein adsorption, induce structural changes, and promote cellular uptake. Simultaneously, inflammatory responses are reduced due to altered protein composition, including decreased immunoglobulins and complement proteins and increased regulatory proteins. These findings provide critical insights for understanding the bio‐fate of nanomedicines.

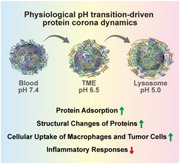

## 1 Introduction

Nanomedicines promise precise drug delivery with minimized side effects. However, their clinical translation is hindered by complex interactions with biological systems.^[^ ^1^, ^2^, ^3^, ^4^ ^]^ Indeed, upon intravenous administration, nanoparticles (NPs) form a “protein corona” (PC) by adsorbing plasma proteins, altering their “chemical identity” to form a new “biological identity”, which then determines their in vivo fate.^[^ ^5^ ^]^ This corona can trigger recognition by the mononuclear phagocyte system (MPS) through opsonin adsorption (e.g., complement proteins, immunoglobulins), extend circulation time via non‐opsonin adsorption (e.g., apolipoproteins),^[^ ^6^, ^7^, ^8^, ^9^, ^10^, ^11^ ^]^ and mask targeting ligands to diminish targeting efficacy.^[^ ^12^ ^]^ Furthermore, interactions with NPs can induce conformational changes of proteins,^[^ ^13^ ^]^ thus affecting their functions,^[^ ^14^ ^]^ exposing hidden binding sites to trigger unexpected receptor‐specific recognition,^[^ ^15^ ^]^ altering cellular internalization pathways,^[^ ^16^ ^]^ and activating immune responses, etc.^[^ ^17^, ^18^, ^19^ ^]^ Therefore, revealing protein conformation changes is crucial for understanding how the PC affects the biological fate of NPs.^[^ ^20^ ^]^ While physical and surface chemistry of NPs are known to influence protein conformation and immune recognition,^[^ ^18^, ^21^ ^]^ the effects of changes in physiological environments on PC evolution remain underexplored.

The composition of PC is highly dependent on the local physiological microenvironment. Studies show that variations in body fluid composition can induce dynamic changes in the PC. For instance, intracellular proteins like pyruvate kinase M2 (PKM2) and chaperone proteins can replace initially adsorbed blood proteins as NPs traverse from the bloodstream into lysosomes and cytoplasm.^[^ ^22^ ^]^ This replacement disrupts proteostasis and triggers chaperone‐mediated autophagy (CMA), ultimately interfering with key metabolic pathways such as glycolysis and lipid metabolism. Recently, Miao et al.^[^ ^23^ ^]^ reported that during nanovesicle transport from blood to tumors, glycosylated nanovesicles dynamically adsorb tumor‐associated proteins, such as CD44 and osteopontin, which have low isoelectric points. These proteins bind to the amino groups of glycosylated nanovesicles, enhancing selective uptake by tumor cells. Moreover, as NPs pass through damaged blood vessels, they may encounter a high concentration of platelet factor 4 proteins released by activated platelets to form a transient protein corona. These bound platelet factors can interact with heparan sulfate proteoglycans on endothelial cells and promote cellular uptake.^[^ ^24^ ^]^ Nevertheless, the effects of physiological conditions, such as pH values, oxygen levels, glycemic concentrations, and reductive/oxidative states, etc., on the PC evolution remain largely unexplored. Among them, acidic pH is a hallmark of the tumor microenvironment and has been widely leveraged to design smart nanomedicines with tumor‐selective drug release profiles.^[^ ^25^, ^26^, ^27^ ^]^ During in vivo delivery to tumor cells, NPs undergo a series of pH transitions: starting in the bloodstream (pH 7.4), transitioning into the mildly acidic tumor microenvironment (pH 6.5), and eventually reaching intracellular compartments such as lysosomes (pH 4.5–5.5).^[^ ^27^, ^28^, ^29^ ^]^ These pH shifts can significantly alter protein charges, induce conformational changes, and even result in exposure of new binding sites,^[^ ^30^, ^31^, ^32^ ^]^ thereby playing a crucial role in shaping the PC composition and influencing nanoparticle interactions with cells. Additionally, inflammatory sites and hypoxic necrotic regions can create localized acidic environments,^[^ ^33^ ^]^ while metabolic disorders such as diabetic ketoacidosis^[^ ^34^ ^]^ and chronic kidney^[^ ^35^ ^]^ disease can lead to systemic blood pH reductions approaching neutrality. Thus, elucidating the effects of physiological pH transitions on the PC fingerprinting and conformation is critical for understanding the complex in vivo behaviors of nanomedicines.

**Figure 1:**
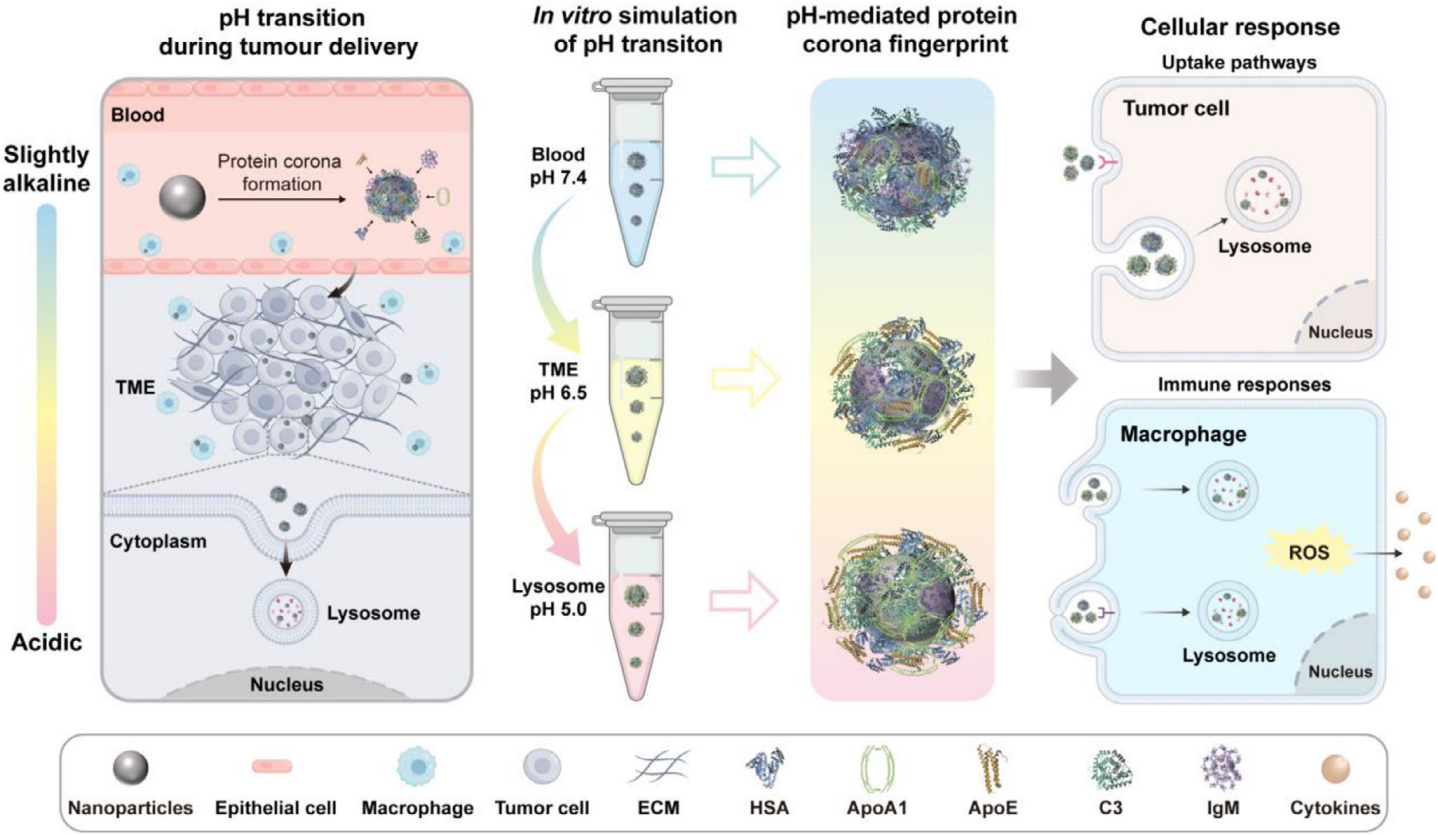
Illustration of the impact of changes in physiological pH on the evolution of protein corona on nanoparticles, and in turn on cellular uptake and inflammatory responses during tumor therapy.

In this study, we investigated how physiological pH transitions influence the evolution of protein corona on silica‐coated magnetic nanoparticles (SMNs) and their subsequent biological effects (**Figure** **1**). SMNs were incubated with human serum (HS) to form protein corona‐coated SMNs (PC@SMNs), which were then exposed to different pH environments to simulate the pH transitions encountered during tumor delivery (Scheme S1, Supporting Information). We systematically investigated PC compositions and protein conformation changes under different pH conditions. Subsequently, the effect of pH‐induced PC changes on cellular uptake, internalization mechanisms, and intracellular trafficking of SMNs was studied on macrophages and tumor cells. The induced inflammatory responses were investigated with macrophages by detecting reactive oxygen species (ROS) levels and the secretion of inflammatory cytokine (IL‐1*β*, TNF‐*α*, and IL‐6). Hence, we established the correlation between pH values, PC fingerprinting, and biological behaviors of NPs, revealing that microenvironmental pH affects the composition and protein conformation of PC, which in turn impacts the biological profiles of NPs. This study underscores the essential role of physiological pH transitions in regulating biological behavior and the therapeutic efficacy of nanomedicines.

## 2 Results and Discussion

### 2.1 Preparation and Characterization of SMNs and PC@SMNs

SMNs were synthesized following reported methods^[^ ^36^ ^]^ and characterized using transmission electron microscopy (TEM) and dynamic light scattering (DLS), showing uniform spherical nanoparticles (Figure S1a, Supporting Information) with a hydrodynamic diameter (*D*_h_) of 134 ± 1 nm (Figure S1b, Supporting Information). The discrepancy between TEM (≈50 nm core size) and DLS (≈130 nm) results arises naturally from the measurement principle: DLS captures the hydrated diameter (including water shell), while TEM measures the dry‐state solid core. Moreover, the intensity‐weighted hydrodynamic diameter from DLS measurement is strongly influenced by larger particles, since scattering intensity scales with the sixth power of particle diameter (*I ∝ d*⁶).^[^ ^37^ ^]^ To further validate the particle size in a hydrated state with higher resolution, we employed nano‐flow cytometry (nanoFCM) that provides accurate nanoparticle sizing comparable to TEM. The average size of SMNs measured by nanoFCM was 60.8 ± 16.7 nm (Figure S1c, Supporting Information), aligning closely with the core diameter observed by TEM.

**Figure 2:**
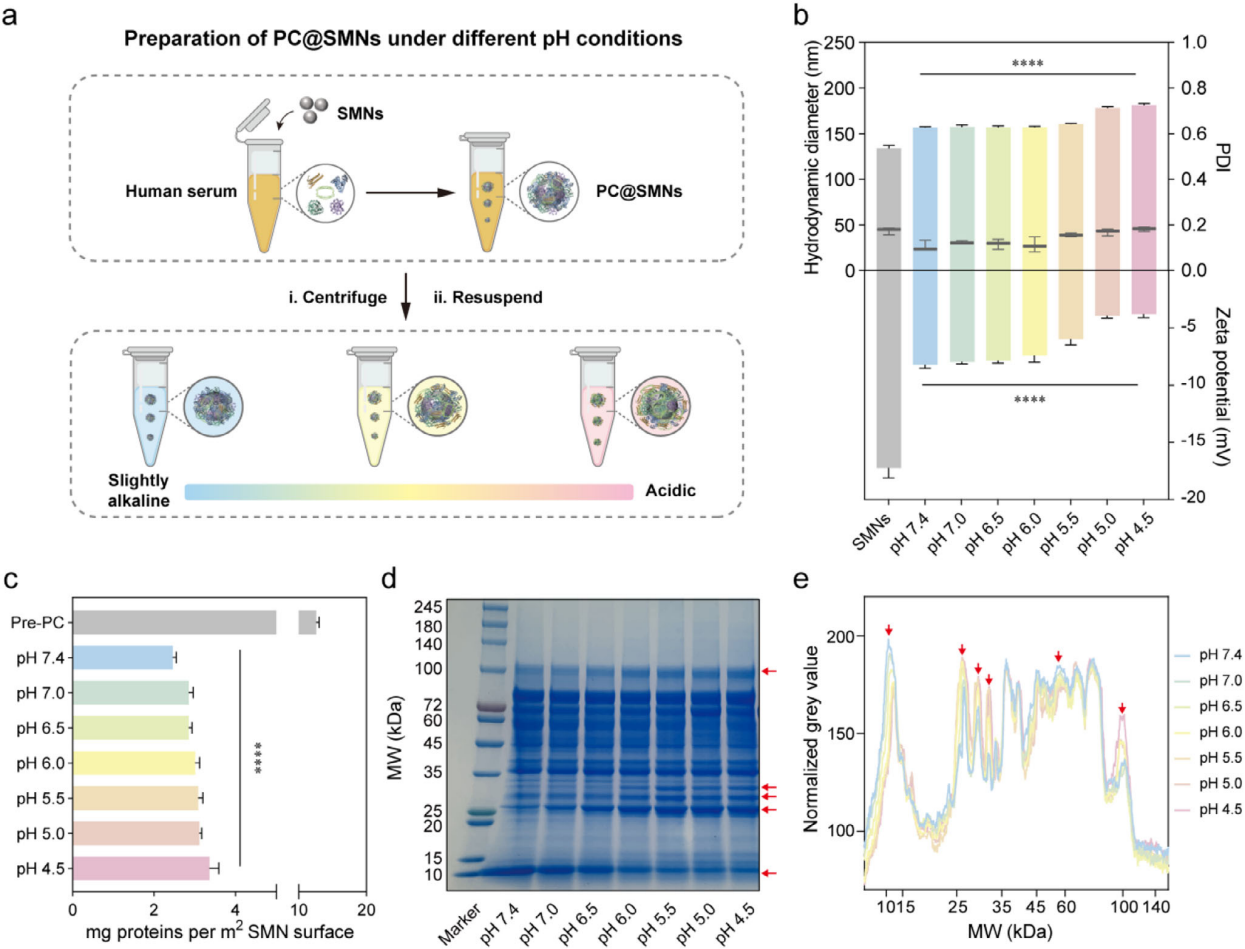
Characterization of PC@SMN formation at different pH values. a) Schematic illustration of PC@SMN formation under different pH conditions. b) *D*_h_, PDI, and zeta potential of SMNs and PC@SMNs. c) Protein quantification of PC@SMNs using the BCA assay. Data are presented as mean ± standard deviation with *n* = 3 (biologically independent experiments). Statistical significance was tested with ordinary one‐way ANOVA, *p < 0.05, **p < 0.01, ***p < 0.001, and ****p < 0.0001. d) Coomassie‐stained SDS‐PAGE gel showing the protein composition of PC@SMNs. MW, molecular weight. e) Densitometric analysis of Coomassie‐stained SDS–PAGE gel of PC@SMNs exposed to different pH conditions in (d). Red arrows indicate protein bands exhibiting marked intensity changes across different pH conditions.

To simulate protein corona formation in the bloodstream, SMNs were incubated with human serum to generate pre‐formed protein corona‐coated SMNs (Pre‐PC@SMNs). To mimic the pH‐transitioning microenvironments that NPs may encounter under various pathological conditions, Pre‐PC@SMNs were further incubated in PBS buffers ranging from pH 7.4 to 4.5 (**Figure** **2**a). The resulting PC@SMNs exhibited slightly larger *D*_h_ (≈150–180 nm) and lower zeta potentials (−8.0 – −3.0 mV) compared to bare SMNs (Figure 2b), which might be due to protein adsorption from the serum.^[^ ^38^ ^]^ TEM imaging confirmed the presence of protein coronas on PC@SMNs under different pH conditions (Figure S3, Supporting Information). Additionally, the protein corona substantially improved the colloidal stability of SMNs in acidic environments compared to bare SMNs (Figures S2 and S3, Supporting Information). At early time points (1–2 h), *D*_h_ remained stable (≈150–160 nm) from pH 7.4 to 5.5 and increased moderately to ≈180 nm at pH 5.0 and 4.5 (Figure S4a, Supporting Information). This corresponds to a ≈50 nm increase over bare SMNs, consistent with the formation of a protein layer ≈10–25 nm thick on each side. The particles maintained low PDI values (<0.2; Figure S4b, Supporting Information) and unimodal size distributions without secondary peaks (Figure S4c—f, Supporting Information), indicating excellent colloidal stability. Upon prolonged incubation (12–24 h), *D*_h_ remained stable under pH 7.4–6.0 while increasing slightly to ≈190–210 nm at pH < 5.0 (Figure S4a, Supporting Information). This pH‐dependent increase in *D*_h_ can be attributed to the reduced electrostatic repulsion near the isoelectric points of many serum proteins (pH 5.0), which lowers surface charge density and promotes protein retention on SMNs (Figure 2b).^[^ ^32^, ^39^ ^]^ Simultaneously, acid‐induced partial unfolding of protein tertiary structures may expose hydrophobic domains, enhancing protein‐nanoparticle interactions via non‐covalent forces such as hydrophobic interactions, hydrogen bonding, and van der Waals forces.^[^ ^40^, ^41^, ^42^ ^]^ These effects collectively contribute to progressive corona thickening under acidic conditions.

Protein retention was further quantified via BCA assay (Figure 2c), which showed a consistent increase in adsorbed protein with decreasing pH. Spearman correlation analysis (Figure S5, Supporting Information) revealed a strong positive correlation between protein adsorption and *D*_h_ across all time points (*ρ* > 0.88, *p* < 0.05). Notably, Pre‐PC@SMNs were obtained after only a single ultracentrifugation, which still retains a considerable fraction of loosely bound proteins (soft corona) in addition to the tightly bound hard corona.^[^ ^43^ ^]^ Following incubation in plain buffer at different pH levels and a second ultracentrifugation, most of these residual soft corona proteins were removed (Figure 2c). In comparison, the effect of pH on the total amount of retained proteins is relatively small compared with the reduction caused by centrifugation. This observation aligns with the widely accepted two‐layer model of the protein corona, consisting of an inner hard corona and a dynamic, easily removable soft corona.^[^ ^43^ ^]^ The above results indicate that the *D*_h_, zeta potential, and protein retention amount of PC@SMNs are influenced by the pH of local environments.

To investigate the differences in the composition of proteins on PC@SMNs exposed to different pH values, we separated the serum proteins in the corona by sodium dodecyl sulfate‐polyacrylamide gel electrophoresis (SDS‐PAGE). As shown in Figure 2d–e, variations in environmental pH resulted in slight differences in the abundance of specific proteins (indicated by red arrows). Specifically, as pH decreased, the relative intensity of protein bands with molecular weights of 100 kDa and 25–35 kDa gradually increased, while the relative abundance of protein bands around 10 kDa decreased, indicating that the composition of protein corona changes upon environmental pH evolution.

### 2.2 LC‐MS/MS Analysis of PC Compositions Exposed to Different pH Values

**Figure 3:**
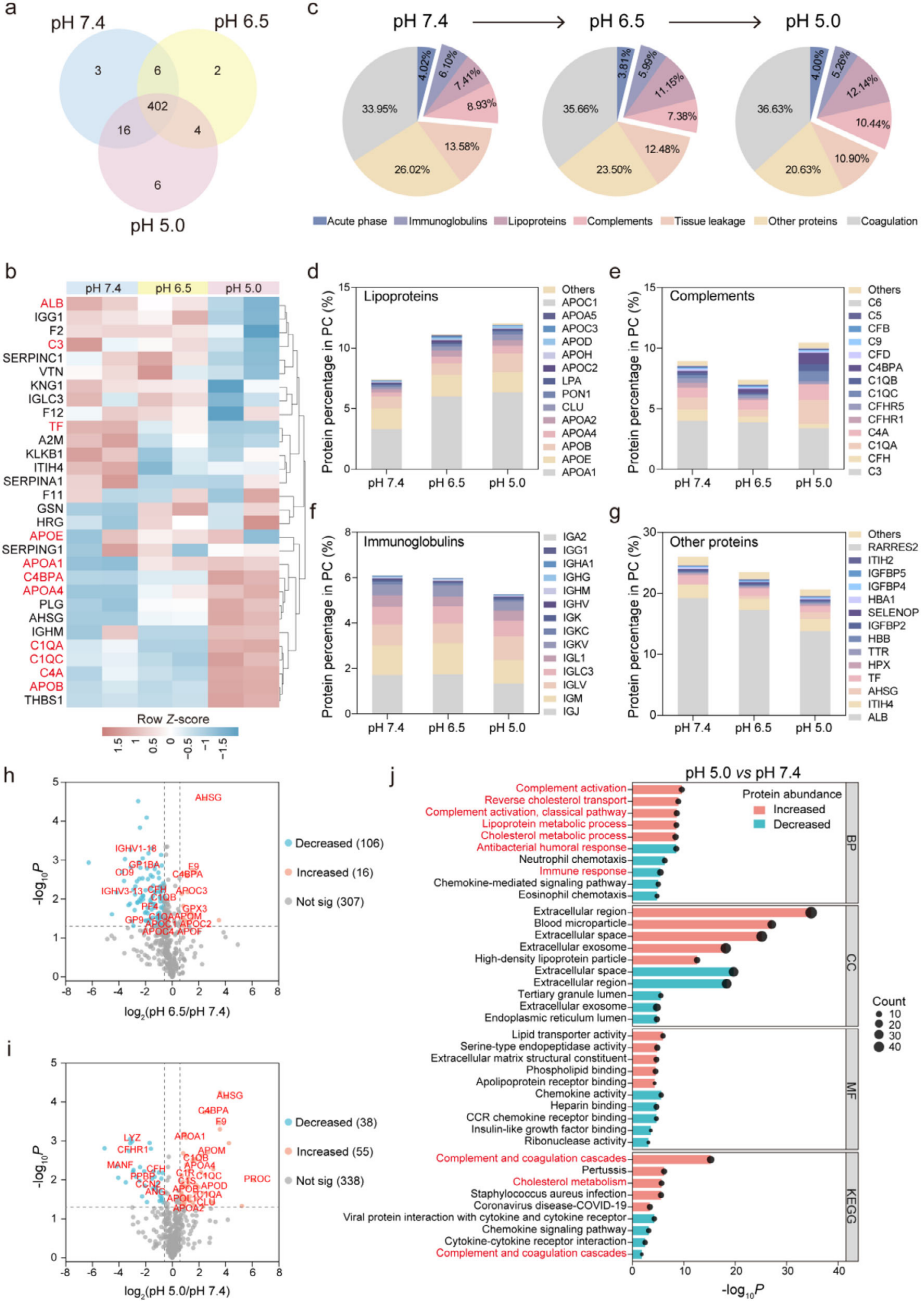
Proteomics analysis of the PC@SMNs exposed to different pH values. (a) Venn diagram of overlapped proteins for PC@SMNs. b) Heat map of the top 30 abundant proteins in PC@SMNs with clustering analysis. c) Relative abundance and classification of proteins identified in PC@SMNs, categorized into 6 types based on biological functions. d–g) Relative abundance of the identified lipoproteins (d), complements (e), immunoglobulins (f), and other proteins (g) in PC@SMNs. Data are presented as mean with *n* = 2 (biologically independent experiments). h–i) Volcano plot of the protein abundance fold change of (log_2_(pH 6.5/pH 7.4)) (h) and (log_2_(pH 5.0/pH 7.4)) (i) versus statistical significance (−log_10_ *P*). Proteins with increased abundance are shown in red, proteins with decreased abundance in blue. The horizontal dashed line represents *P* = 0.05. The vertical dashed lines represent a fold change of 2/3 and 3/2. j) GO and KEGG analyses of proteins with significantly altered abundance in PC@SMNs (pH 5.0) compared to PC@SMNs (pH 7.4). GO, Gene Ontology; BP, biological processes; CC, cellular components; MF, molecular functions; KEGG, Kyoto Encyclopedia of Genes and Genomes.

To analyze the protein corona fingerprints exposed to different pH conditions, we performed liquid chromatography‐tandem mass spectrometry (LC‐MS/MS) on PC@SMNs prepared at pH 7.4, 6.5, and 5.0, simulating the pH values in blood, tumor tissue, and lysosomal microenvironments encountered during tumor delivery.^[^ ^27^, ^28^ ^]^ As shown in **Figure** **3**a,b, while the types of proteins in all three PC@SMNs remained identical, their relative abundance varied significantly. The identified proteins were categorized into six groups according to their physiological functions (Figure 3c). Coagulation proteins were the most abundant, with their levels increasing as environmental pH decreased. This was followed by tissue leakage proteins, complement proteins, lipoproteins, immunoglobulins, and acute phase proteins (Figure 3c; Figure S6, Supporting Information). Notably, the abundance of lipoproteins, primarily ApoA1, ApoE, and ApoB, increased with decreasing pH (Figure 3d). These apolipoproteins are known to mediate cellular uptake of NPs via receptor‐mediated endocytosis.^[^ ^44^, ^45^ ^]^ Among the complement proteins, C3 was the most abundant, and its level decreased as environmental pH was lowered (Figure 3e). As a key component of the complement system, the activation of C3 initiates a cascade that promotes pathogen recognition and phagocytosis.^[^ ^46^, ^47^ ^]^ Immunoglobulin content decreased with decreasing pH values (Figure 3e). The abundance of immunoglobulins, which act as opsonins facilitating NPs’ clearance by macrophages, also diminished at lower pH levels.^[^ ^48^, ^49^, ^50^, ^51^ ^]^ Albumin, the most abundant protein in human serum and known to reduce opsonization and mitigate NP‐induced inflammation,^[^ ^52^ ^]^ was highly prevalent in the corona, with its concentration increasing as the pH dropped (Figures 3d,g).

Compared to PC@SMNs exposed to pH 7.4, 16 proteins exhibited increased abundance while 106 proteins exhibited decreased abundance upon PC@SMNs exposed to pH 6.5 (Figure 3h), while 55 proteins showed increased abundance and 38 proteins showed decreased abundance PC@SMNs exposed to pH 5.0 (Figure 3i). These differentially abundant proteins include the complement proteins (C3, C4BPA, C1QA, C1QC, and C4A) and lipoproteins (ApoE, ApoA1, ApoA4, and ApoB) (Figure 3b). Gene Ontology (GO) and KEGG pathway analyses also revealed that these differentially abundant proteins are mainly involved in complement activation and cholesterol metabolism pathways (Figures 3j; Figure S7, Supporting Information). Overall, the above results suggest that the environmental pH influences the protein composition of PC@SMNs, likely due to pH‐induced structural and hydrophobicity changes that affect protein binding affinities to SMNs.

### 2.3 Analysis of SMN‐HSA Interactions at Different pH Values

**Figure 4:**
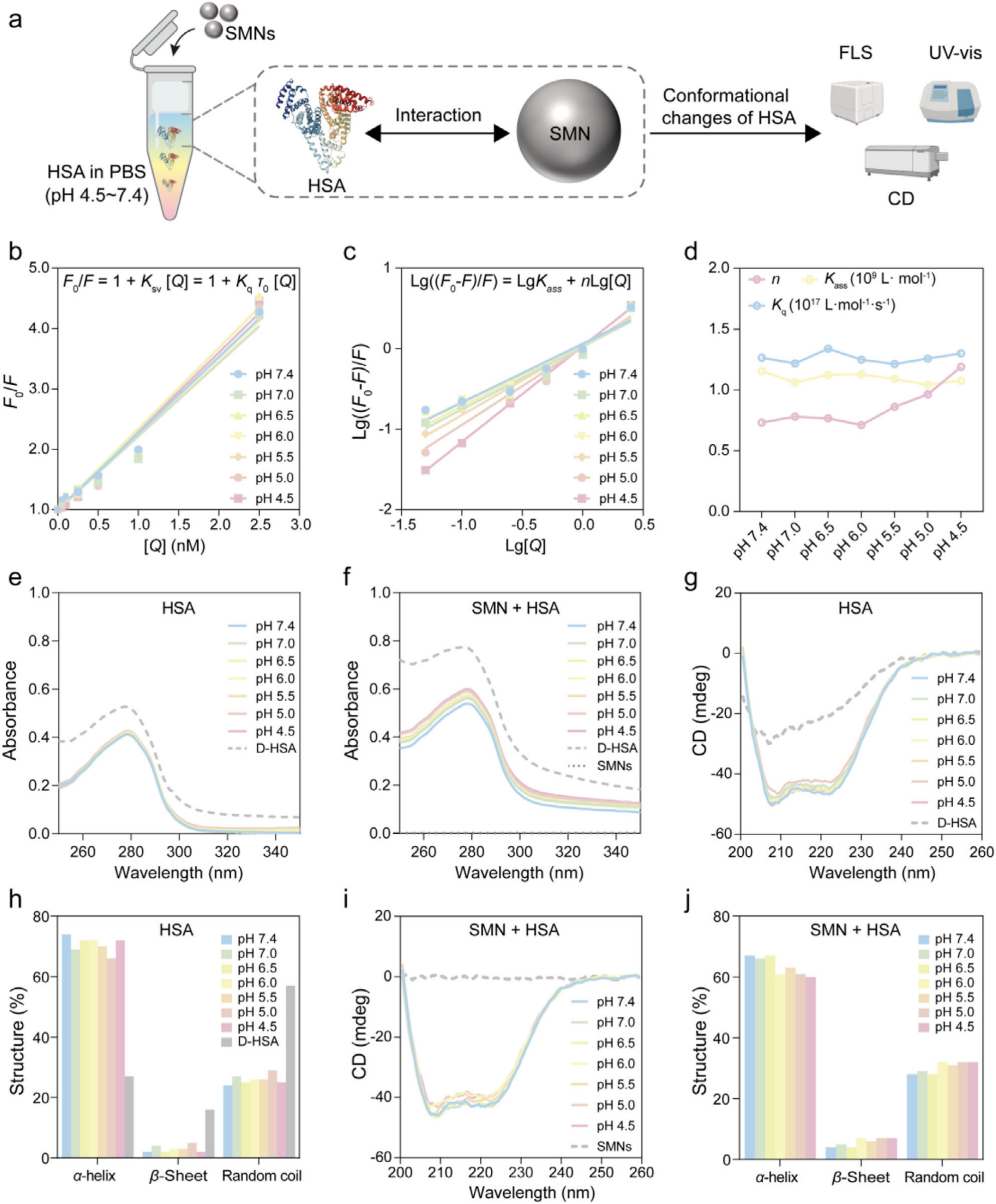
Interactions between HSA and SMNs at different pH values. a) Schematic illustration of the interactions between HSA and SMNs under varying pH conditions, characterized by fluorescence, UV–vis, and CD spectroscopies. The FLS, UV–vis, and CD instrument images are from BioRender.com. b) Fluorescence quenching (Stern‐Volmer plots) and c) Fluorescence quenching (Scatchard plots) of HSA after interaction with SMNs under different pH conditions. d) *K*_q_, *K*_ass_, and *n* values for HSA‐SMN interactions under different pH conditions. e,f) UV–vis spectra of HSA in the absence (e) and presence (f) of SMNs under different pH conditions. Data are presented as mean with *n* = 3 (independent measurements). g–j) CD spectra and secondary structure analysis (e.g., *α*‐helix, *β*‐sheet, and random coil) of HSA in the absence (g‐h) and presence (i‐j) of SMNs under different pH conditions.

To investigate the effect of environmental pH on protein structure changes, we selected HSA as a model protein, given its high abundance in both human plasma and protein corona of SMNs,^[^ ^53^ ^]^ and the structure of HSA after interaction with NPs can affect the biological fate of NPs by regulating nanoparticle‐receptor interactions,^[^ ^15^ ^]^ cellular uptake,^[^ ^21^, ^54^ ^]^ immune recognition,^[^ ^55^ ^]^ and inducing inflammatory responses.^[^ ^56^ ^]^ Fluorescence spectrometer (FLS), ultraviolet‐visible (UV‐vis) spectrophotometer, and circular dichroism (CD) spectrometer were employed to study the interactions between HSA and SMNs under various pH conditions (**Figure** **4**a).

Using FLS, we measured the fluorescence spectra of HSA before and after exposure to various concentrations of SMNs (Figure S4, Supporting Information). HSA contains two tryptophan residues (Trp‐134 and Trp‐212) that contribute to its intrinsic fluorescence.^[^ ^57^, ^58^ ^]^ As shown in Figure S8 (Supporting Information), heat‐denatured HSA (D‐HSA) exhibited significantly reduced fluorescence intensity compared to native HSA, suggesting a loss of intrinsic fluorescence upon denaturation.^[^ ^59^ ^]^ Before exposure to SMNs, the maximum fluorescence intensity of HSA slightly decreased as the pH decreased, indicating slight protein unfolding at low pH.^[^ ^32^ ^]^ However, after interacting with SMNs, the fluorescence intensity of HSA markedly decreased with increasing SMN concentrations (10 µg mL^−1^ to 500 µg mL^−1^) across all pH conditions (Figure S8b‐h). This fluorescence quenching indicated a strong interaction between HSA and SMNs.^[^ ^60^ ^]^ At the same SMN concentrations, HSA's fluorescence intensity further decreased as pH was lowered, implying that acidic conditions enhance HSA‐SMN interactions, leading to larger unfolding of HSA.

To elucidate the quenching mechanism induced by SMN‐HSA interactions, the data were analyzed using the Stern‐Volmer equation, as shown in Equation (1):^[^ ^61^, ^62^ ^]^

(1) $$\frac{F_{0}}{F}=1+K_{\mathrm{sv}}\left[Q\right]=1+K_{q}\tau_{0}\left[Q\right]$$

where *F*_0_ and *F* are the fluorescence intensities of HSA in the absence and presence of SMNs, respectively, *K*_sv_ is the Stern‐Volmer quenching constant, *K*_q_ is the bimolecular quenching rate constant, *τ*_0_ is the average fluorescence lifetime of the biomolecule (≈10^−8^ s), and [*Q*] is the concentration of SMNs incubated with HSA. The plot of $\frac{F_{0}}{F}$ versus [*Q*] showed a good linearity under all pH conditions (Figure 4b), suggesting a single quenching mechanism, either dynamic or static. For static quenching, where a complex forms between the quencher and the biomolecule, the *K*_q_ value exceeds 2.0 × 10^10^ L·mol^−1^·s^−1^. As shown in Figure 4d, all calculated *K*_q_ values were over this threshold, indicating a static quenching mechanism due to interaction between HSA and SMNs.

The binding molar ratio, binding constant (*K*_ass_), and the number of binding sites (*n*) for HSA was determined using the Scatchard plot analysis, as shown in Equation (2):^[^ ^63^ ^]^

(2) $$\mathrm{lg}\left(\frac{F_{0}-F}{F}\right)=\;\mathrm{lg}K_{\mathrm{ass}}+n\mathrm{lg}\left[Q\right]$$

where lg[Q] is the logarithm of the quencher concentration and $\mathrm{lg}(\frac{F_{0}-F}{F})$ is the logarithm of the fluorescence intensity ratio in the absence and presence of SMNs. Linear regression allowed the calculation of *K*_ass_ from the intercept and *n* from the slope (Figure 4c). The results showed that while *K*_ass_ slightly decreased as pH dropped, *n* significantly increased, indicating that acidic pH strengthened HSA‐SMN interactions, leading to greater unfolding of HSA and increased contact area with SMNs.

To further investigate the conformational changes of protein upon interaction with SMNs, we employed UV‐vis spectroscopy to analyze the absorption spectra of HSA before and after incubation with SMNs. The UV absorption peak of HSA at 278 nm arises from its aromatic amino acids (Trp, Tyr, and Phe).^[^ ^64^ ^]^ As shown in Figure 4e, the absorbance of D‐HSA at 278 nm was significantly higher than that of HSA at pH 7.4, indicating increased aromatic residue exposure caused by protein unfolding. In the absence of SMNs, HSA exhibited negligible absorbance changes across pH 7.4–4.5, indicating minimal structural perturbation induced by pH alone. This observation is consistent with previous reports showing that HSA retains its normal (N) form from pH 9.0 to 4.5, transitions to a fast (F) form at pH 4.0–3.5, and becomes fully extended (E) form below pH 3.0.^[^ ^30^, ^65^ ^]^ In contrast, upon interaction with SMNs, the absorbance of HSA at 278 nm increased progressively as pH decreased (Figure 4f), and this trend became more pronounced from pH 7.4 to 4.5 (Figure S9, Supporting Information), indicating that HSA‐SMN interactions significantly promote protein unfolding, particularly at lower pH values. This effect is likely attributable to the fact that pH 5.0 is close to the isoelectric point of HSA, which leads to a reduction in net protein charge and thereby weakens the electrostatic repulsion between HSA and the negatively charged SMNs. The resulting decrease in repulsive forces enhances protein‐nanoparticle affinity, facilitating stronger and more stable adsorption. These interactions, in turn, promote tertiary structure destabilization by disrupting the native hydrophobic core, and increasing the exposure of buried aromatic residues. This observation aligns with the FLS results, showing that acidic environments intensify SMN‐induced changes in HSA conformation.

CD spectroscopy was employed to analyze the secondary structure changes of HSA upon interaction with SMNs.^[^ ^66^ ^]^ The CD spectrum of free HSA typically exhibits two negative peaks at 208 and 222 nm, corresponding to the *α*‐helix structure and the n‐π* electronic transition, respectively.^[^ ^67^, ^68^ ^]^ In our study, the CD spectrum of HSA displayed two negative peaks at 208 nm and 222 nm (Figure 4g), while SMNs themselves did not exhibit any circular dichroism, therefore showing no discernible absorption peaks (Figure 4i). Upon denaturation, HSA exhibited a disrupted secondary structure with a reduction in *α*‐helix content and an increase in *β*‐sheet and random coil content, as seen in the D‐HSA (Figure 4h). Before interacting with SMNs, the *α*‐helix, *β*‐sheet, and random coil contents of HSA remained relatively stable across varying pH values (Figure 4h). However, after exposure to SMNs (Figure 4j), the *α*‐helix content of HSA decreased from ≈72% to ≈65%, while the *β*‐sheet content increased from ≈3% to ≈7% while the random coil content increased from ≈25% to ≈32%. Furthermore, as the pH decreased, a further decrease in *α*‐helix content was observed (from ≈67% to ≈60%), alongside a slight increase in *β*‐sheet (from ≈4% to ≈7%) and random coil (from ≈28% to ≈32%) contents. These findings suggest that the interaction between SMNs and HSA leads to the unfolding of HSA, with the effect becoming more pronounced as environmental pH decreases. This may be due to a reduction in the charge density of HSA at lower pH, which enhances the interaction with SMNs, thereby promoting further structural perturbations. However, the slight reduction in *α*‐helix content indicates that HSA mainly undergoes tertiary structural changes, rather than significant disruption of secondary structure (i.e., complete denaturation).

### 2.4 Cellular Uptake of PC@SMNs Exposed to Different pH Values

Next, we studied the cellular uptake of PC@SMNs exposed to different pH conditions by macrophages and tumor cells.^[^ ^3^, ^4^ ^]^ RAW264.7 and differentiated macrophage‐like THP‐1 (dTHP‐1) cells were used as macrophage models, and A549 cells served as tumor cell model. Prior to the uptake study, we evaluated the cytocompatibility of SMNs (Figure S10, Supporting Information). Within the SMN concentration range of 0–250 *µ*g mL^−1^, cell viability of RAW264.7, dTHP‐1, and A549 cells remained above 80%, demonstrating the good biocompatibility of SMNs at these concentrations.

**Figure 5:**
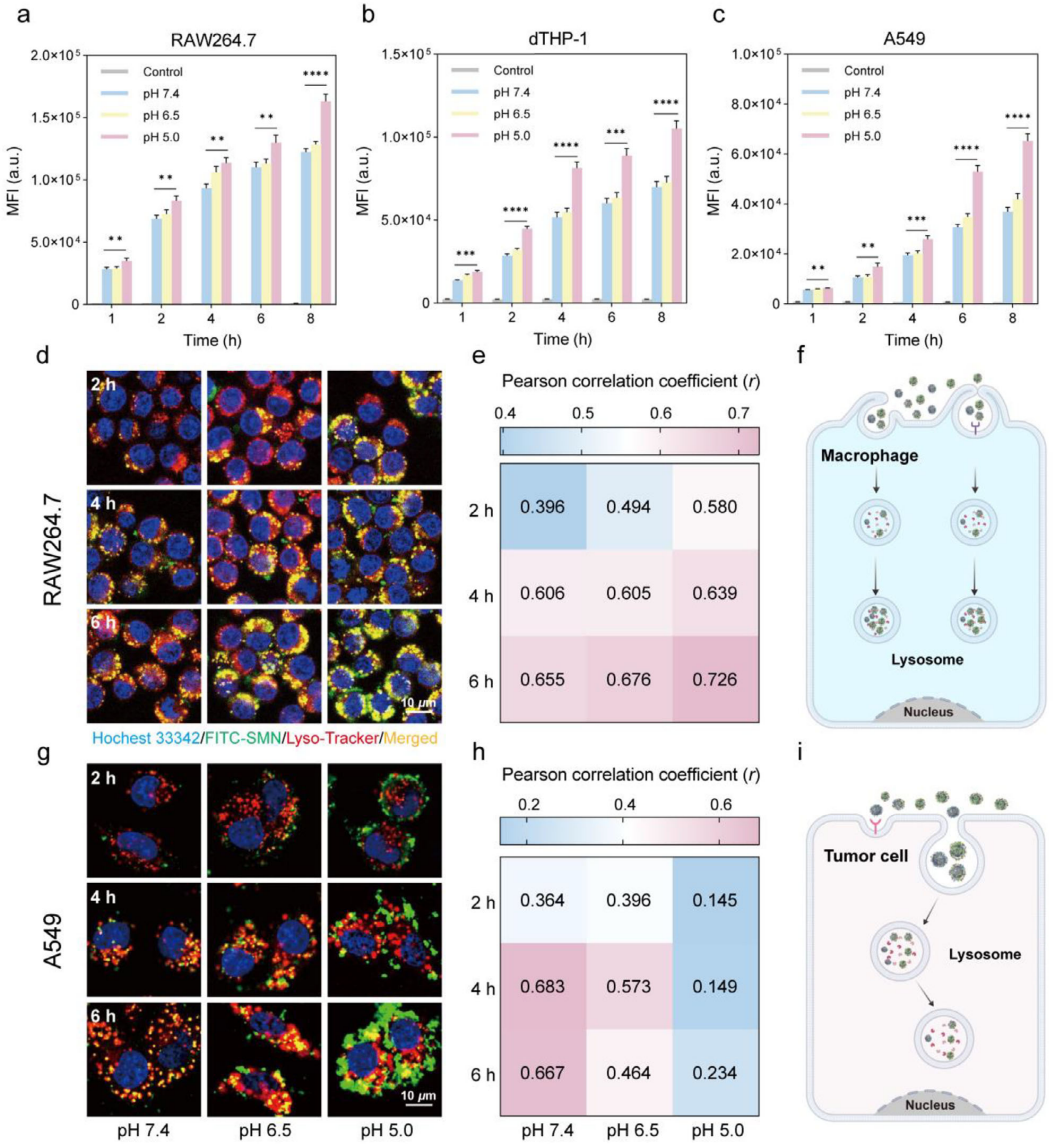
Cellular uptake and intracellular trafficking of PC@SMNs exposed to different pH values. a–c) FCM analysis of PC@SMN uptake by RAW264.7 (a), dTHP‐1 (b), and A549 (c) cells in serum‐free medium for 1 h, 2 h, 4 h, 6 h, and 8 h. Data are presented as mean ± standard deviation with *n* = 3 (biologically independent experiments). Statistical significance was tested with ordinary one‐way ANOVA, *p < 0.05, **p < 0.01, ***p < 0.001, and ****p < 0.0001. d) CLSM images showing the intracellular localization of PC@SMNs in RAW264.7 cells in serum‐free medium. SMNs were labelled with FITC (green); nuclei were stained with Hoechst 33 342 (blue); lysosomes were stained with Lyso‐Tracker Red. Scale bars: 20 *µm*. e) Pearson correlation coefficients (*r*) for FITC and Lyso‐Tracker Red fluorescence co‐localization in RAW264.7 cells. f) Schematic illustration of cellular uptake and intracellular trafficking of PC@SMNs exposed to different pH conditions in macrophage cells: macrophage uptake of PC@SMNs increased over time, with the PC@SMNs predominantly localized in lysosomes. g) CLSM images showing the intracellular localization of PC@SMNs in A549 cells in serum‐free medium. SMNs were labelled with FITC (green); nuclei were stained with Hoechst 33342 (blue); lysosomes were stained with Lyso‐Tracker Red. Scale bars: 20 *µm*. h) Pearson correlation coefficients (*r*) for FITC and Lyso‐Tracker Red fluorescence co‐localization in A549 cells. i) Schematic illustration of cellular uptake and intracellular trafficking of PC@SMNs exposed to different pH conditions in tumor cells: tumor cell uptake of PC@SMNs increased with exposure time, initially localizing in lysosomes before escaping into the cytoplasm. Data are presented as mean with *n* = 3 (biologically independent experiments).

Cellular uptake of PC@SMNs was assessed across different cell types using flow cytometry (FCM) over a time course of 1 to 8 h (**Figure** **5**a–c). In serum‐free medium, the uptake of PC@SMNs by RAW264.7, dTHP‐1, and A549 cells exhibited a time‐dependent increase and reached near‐saturation around 2 h, with the percentage of positive cells approaching 100% (Figure S11, Supporting Information). In addition, at each time point, PC@SMNs (pH 5.0) had a higher cell uptake, indicating that the protein corona formed in the acidic environment promoted the cell uptake of nanoparticles. Confocal laser scanning microscopy (CLSM) further confirmed enhanced internalization at lower pH values across all cell lines (Figure S12, Supporting Information). This pH‐dependent variation in cellular uptake can be attributed to the increase in *D*_h_ and zeta potential of PC@SMNs exposed to lower pH, which reduces electrostatic repulsion between nanoparticles and cell membrane, thereby facilitating uptake (Figure 2b).^[^ ^69^, ^70^ ^]^ Moreover, alterations in the PC composition under acidic conditions may further enhance cellular internalization of PC@SMNs (Figure 3).^[^ ^71^, ^72^, ^73^ ^]^

We further investigated the correlation between cellular uptake of PC@SMNs exposed to different pH conditions and their PC compositions to identify the key proteins influencing uptake. Pearson correlation coefficients (*r*) revealed that complement proteins, coagulation proteins, immunoglobulins, and lipoproteins are strongly correlated with cell uptake (Figure S13a–c, Supporting Information). For RAW264.7 cells, C4BPA (complement component four binding protein *α*) showed the highest positive correlation with cell uptake (*r* = 0.9999, *P* = 0.0081) (Figure S13a, Supporting Information). C4BPA can recognize and bind to apoptotic/necrotic cells, thereby facilitating immune clearance.^[^ ^74^, ^75^ ^]^ This suggests that C4BPA‐enriched PC may enhance the uptake of NPs by macrophages, which is consistent with previous studies.^[^ ^51^ ^]^ In contrast, C3 (*r* = −1.000, *P* = 0.0058) and KNG1 (*r* = −1.000, *P* = 0.0039) were negatively correlated with RAW264.7 uptake. As a key protein in the complement system, C3 activation typically triggers a complement cascade response that promotes pathogen recognition and phagocytosis.^[^ ^46^, ^47^ ^]^ Here, C3 concentrations remained consistent across samples prepared under different pH conditions, with only very slight reduction as pH decreased. Hence, C3 is excluded as a major factor modulating cell uptake, despite its strong negative correlation with RAW264.7 uptake. For dTHP‐1 cells, ApoB, a key component of low‐density lipoprotein (LDL), was highly correlated with uptake, probably due to its binding to LDL receptors on dTHP‐1 cells (*r* = 1.000, *P* = 0.0057) (Figure S13b, Supporting Information).^[^ ^76^, ^77^ ^]^ For A549 cells, cell uptake correlated positively with C4BPA (*r* = 0.9997, *P* = 0.0151) and FN1 (*r* = 1.000, *P* = 0.0059), and negatively correlated with C3 (*r* = −1.000, *P* = 0.0012). In conclusion, cell uptake of PC@SMNs is strongly correlated with specific proteins in the PC, highlighting the crucial role of PC composition in modulating NP‐cell interactions.^[^ ^6^, ^48^, ^71^, ^72^, ^73^ ^]^

### 2.5 Intracellular Trafficking of PC@SMNs Exposed to Different pH Values

To investigate whether pH‐induced changes of PC composition affect the intracellular behavior of PC@SMNs, we examined their subcellular localization after internalization. As shown in Figure 5d, CLSM imaging revealed that PC@SMNs were internalized by RAW264.7 cells and predominantly localized within lysosomes. The colocalization of PC@SMNs with lysosomes was time‐dependent, with the Pearson correlation coefficient (*r*) increasing over time (Figure 5e,f). Notably, PC@SMNs (pH 6.5 and pH 5.0) showed greater colocalization with lysosomes than the PC@SMNs (pH 7.4). This enhanced lysosomal localization may result from protein denaturation (e.g., HSA) (Figure 4), which makes PC@SMNs (pH 6.5 and pH 5.0) more readily recognized by multiple receptors, particularly those mediating lysosomal trafficking pathways (e.g., SR‐A1, LDLR, and SR‐B1). Consistent with previous studies, receptor‐mediated uptake commonly directs nanoparticles to lysosomes.^[^ ^78^, ^79^, ^80^ ^]^

For A549 cells, PC@SMNs (pH 7.4 and pH 6.5) were internalized and initially localized within lysosomes (Figure 5g). The colocalization coefficient (*r*) initially increased and then decreased over time (Figure 5h), suggesting that PC@SMNs (pH 7.4 and pH 6.5) were predominantly localized in lysosomes at 2–4 h, but their proportion in lysosomes decreased at 6 h, which may reflect redistribution among different subcellular compartments or potential translocation to the cytoplasm (Figure 5i). In contrast, PC@SMNs (pH 5.0) showed minimal lysosomal localization, with *r* = 0.234 after 6 h. CLSM images (Figure 5g) revealed significant membrane adhesion of PC@SMNs (pH 5.0), which became more pronounced over time. The enhanced membrane adhesion may be attributed to the low surface charge PC@SMNs exposed to lower pH (Figure 2b), which reduces electrostatic repulsion between nanoparticles and cell membrane. These interactions may hinder NP entry through conventional endocytosis, thereby reducing lysosomal localization.

### 2.6 Uptake Mechanisms of PC@SMNs Exposed to Different pH Values

**Figure 6:**
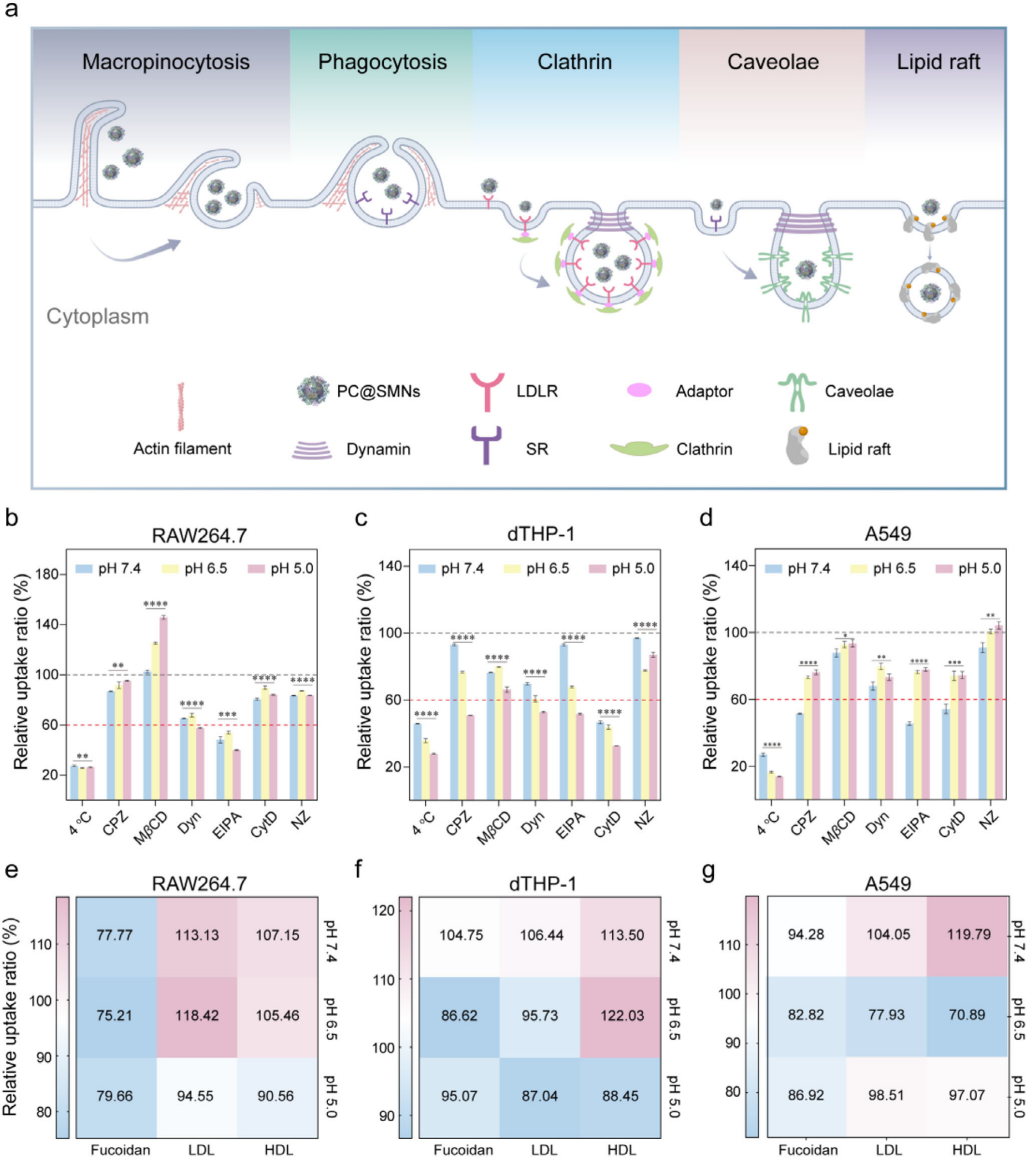
Uptake mechanisms of PC@SMNs exposed to different pH values. a) Schematic illustration of the inhibition of different internalization pathways of PC@SMNs. b–d) Cellular uptake of PC@SMNs by RAW264.7 (b), dTHP‐1 (c), and A549 (d) in serum‐free medium after pretreating the cells with various inhibitors. CPZ, chlorpromazine; EIPA, 5‐(*N*‐ethyl‐*N*‐isopropyl) amiloride; M*β*CD, methyl‐*β*‐cyclodextrin; Dyn, dynasore; CytD, cytochalasin D; NZ, nocodazole. Data are normalized relative to the uptake in cells without inhibitors. Data are presented as mean ± standard deviation with *n* = 3 (biologically independent experiments). Statistical significance was tested with ordinary one‐way ANOVA, *p < 0.05, **p < 0.01, ***p < 0.001, and ****p < 0.0001. The grey dashed line represents 100% uptake; the red dashed line marks 60% uptake, indicating the threshold for inhibition efficacy. e–g) Cellular uptake of PC@SMNs by RAW264.7 (e), dTHP‐1 (f), and A549 (g) in serum‐free medium after pretreatment with fucoidan, LDL, or HDL. Data are normalized relative to the uptake in cells without fucoidan, LDL, or HDL pre‐treatment. Data are presented as mean with *n* = 3 (biologically independent experiments).

To explore how pH‐mediated changes in PC composition influence the cellular uptake mechanisms of PC@SMNs, we employed various endocytosis inhibitors (**Figure** **6**a). Chlorpromazine (CPZ) inhibits clathrin‐mediated endocytosis by relocating clathrin and AP2 from the cell surface to intracellular endosomes.^[^ ^81^, ^82^ ^]^ 5‐(*N*‐ethyl‐*N*‐isopropyl) amiloride (EIPA) inhibits macropinocytosis by preventing the formation of actin‐driven membrane ruffles, impairing Na^+^/H^+^ exchanger activity, and blocking extracellular fluid uptake.^[^ ^83^, ^84^, ^85^ ^]^ Cytochalasin D, an actin inhibitor, induces the depolymerization of the actin cytoskeleton, which is crucial for membrane ruffle formation during macropinocytosis. Actin cytoskeleton is also involved in other processes, such as clathrin‐mediated endocytosis.^[^ ^86^, ^87^, ^88^ ^]^ Nocodazole is a microtubule transport inhibitor involved in inhibiting micropinocytosis.^[^ ^89^ ^]^ Dynasore inhibits dynamin, a protein mediating cell membrane scission to form endosomes and essential for multiple endocytic pathways, including clathrin‐mediated endocytosis and other dynamin‐dependent mechanisms.^[^ ^90^, ^91^ ^]^ Methyl‐*β*‐cyclodextrin (M*β*CD) removes cholesterol from the cell membrane, commonly used to inhibit lipid raft‐mediated mechanisms.^[^ ^16^ ^]^

We first screened non‐toxic concentrations of these inhibitors through cell viability assay (Figures S14–S16, Supporting Information). Next, cells were pre‐incubated with inhibitors for 1 h, followed by co‐incubation with PC@SMNs for 2 h. Cellular uptake was then quantified using FCM (Figure 6b–d). For RAW264.7 cells, dynasore and EIPA significantly reduced uptake (down to approximately 63% and 47%, respectively) (Figure 6b). CPZ, cytochalasin D, and nocodazole had minimal effects (reduction < 20%), while M*β*CD increased uptake by 20–50% likely due to enhanced membrane fluidity from cholesterol depletion, leading to compensatory uptake through other pathways like macropinocytosis. These results indicate that clathrin‐mediated endocytosis and macropinocytosis are the primary pathways of PC@SMN entering RAW264.7 cells. Furthermore, incubation at 4 °C significantly reduced uptake to ≈25%, confirming that PC@SMN uptake by RAW264.7 cells is energy‐dependent. Notably, pH variations influenced uptake inhibition, with the strongest inhibition observed at pH 5.0, which can be attributed to increased protein adsorption at lower pH that facilitates receptor‐mediated recognition and uptake.

In dTHP‐1 cells, the uptake of PC@SMNs exposed to pH 7.4 was primarily driven by lipid raft‐mediated endocytosis, clathrin‐mediated endocytosis, and macropinocytosis, with inhibition by approximately 24% for M*β*CD, 30% for dynasore, and 53% for cytochalasin D (Figure 6c). For PC@SMNs (pH 6.5), inhibition was more pronounced, with rates of ≈23% for CPZ, 20% for M*β*CD, 39% for dynasore, 32% for EIPA, 56% for cytochalasin D, and 22% for nocodazole, suggesting an additional contribution of phagocytosis compared to PC@SMNs (pH 7.4). Notably, for PC@SMNs (pH 5.0) all pathways showed stronger inhibition, indicating that uptake of PC@SMNs exposed to lower pH involved multiple pathways.

For A549 cells, the uptake of PC@SMNs (pH 7.4) primarily involved clathrin‐mediated endocytosis and macropinocytosis, as evidenced by the significant inhibition rates of ≈48% for CPZ, 32% for dynasore, 54% for EIPA, and 46% for cytochalasin D (Figure 6d). For PC@SMNs prepared at pH 6.5 and 5.0, these pathways remained dominant, though inhibition was less pronounced, with reductions not exceeding 26% in any pathway. Overall, we observed that, compared to PC@SMNs (pH 7.4), a broader range of pathways contributed to the cellular uptake of PC@SMNs exposed to acidic conditions, which leads to enhanced uptake, especially in macrophages.

To investigate the effect of PC composition on the interactions between PC@SMNs and cell receptors, we examined the roles of low‐density lipoprotein receptor (LDLR), scavenger receptor class B type 1 (SR‐A1), and scavenger receptor class B type 1 (SR‐B1), all of which are known to mediate PC‐driven cellular uptake of NPs.^[^ ^21^, ^92^, ^93^ ^]^ In competitive blocking experiments, cells were pre‐incubated with fucoidan (a polysaccharide known to bind SR‐A1 and other lectin‐like receptors), LDL (which primarily binds to LDL receptor family, including LDLR, low‐density lipoprotein receptor‐related protein 1 (LRP1), and SR‐B1), or HDL (which primarily interacts with SR‐B1 but also interacts with LRP1),^[^ ^21^, ^92^, ^93^ ^]^ followed by incubation with PC@SMNs. Cellular uptake of PC@SMNs was quantified using FCM (Figure 6e–g).

For RAW264.7 cells, pre‐incubation with fucoidan reduced PC@SMN uptake by ≈20–25% (Figure 6e), indicating that SR‐A1 plays a significant role in the uptake process. Pre‐incubation with LDL and HDL slightly reduced uptake of PC@SMNs (pH 5.0) by ≈5% and ≈10%, respectively. These results indicate that SR‐A1 is the primary mediator for uptake of PC@SMNs exposed to pH 7.4, while uptake of PC@SMNs exposed to pH 5.0 involves SR‐A1, LDLR, and SR‐B1. The predominant role of SR‐A1 in RAW264.7 cell uptake may be attributed to its high membrane expression as well as HSA denaturation at acidic pH (Figure 4), aligning with a previous study showing that denatured albumin promotes NP recognition by SR‐A1.^[^ ^21^ ^]^

For dTHP‐1 cells, pre‐incubation with fucoidan, LDL, or HDL did not reduce the uptake of PC@SMNs (pH 7.4), suggesting that SR‐A1, LDLR, and SR‐B1 are not involved (Figure 6f). For PC@SMNs (pH 6.5), uptake was reduced by approximately 14% with fucoidan and 5% with LDL pre‐incubation, indicating involvement of SR‐A1 and LDLR. For PC@SMNs (pH 5.0), all three receptors (SR‐A1, LDLR, and SR‐B1) contributed to the uptake, as pre‐incubation with fucoidan, LDL, or HDL reduced uptake by approximately 5%, 13%, or 12%, respectively. The differences in receptor involvement are likely related to protein conformational changes induced by pH variations. For PC@SMNs (pH 6.5 and pH 5.0), the significant denaturation of HSA enhances SR‐A1 recognition as discussed above. In addition, for PC@SMNs (pH 5.0), the abundance of HSA decreased significantly (Figure 3g) while the abundance of lipoproteins increased (Figure 3d), promoting further receptor engagement via LDLR and SR‐B1. Thus, PC@SMNs exposed to lower pH conditions enter the cell through multiple receptors, and the selectivity of these receptors is influenced by both PC composition and protein conformational changes.

For A549 cells, SR‐A1 was involved in the uptake of PC@SMNs (pH 7.4 and pH 5.0), while LDLR and SR‐B1 were not (Figure 6g). For PC@SMNs (pH 6.5), however, all 3 receptors (SR‐A1, LDLR, and SR‐B1) mediated uptake, suggesting that the partially unfolded proteins under mildly acidic conditions engaged multiple receptors. In summary, these results indicate that the interactions between PC@SMNs and cell receptors are modulated by PC compositions and protein conformations, which are shaped by the environmental conditions.^[^ ^15^, ^71^, ^72^ ^]^

### 2.7 Inflammatory Responses of Macrophages to PC@SMNs Exposed to Different pH Values

**Figure 7:**
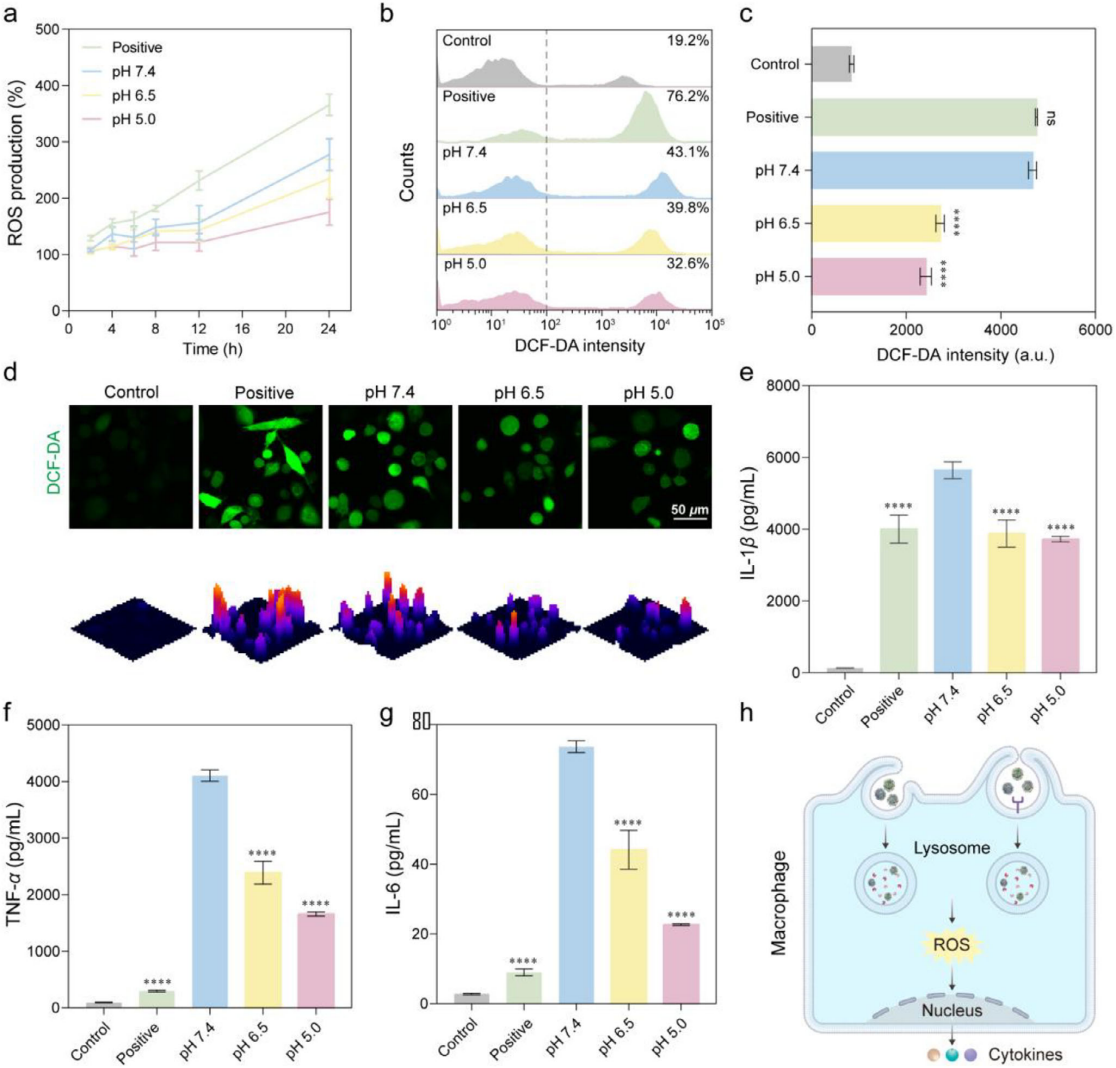
Immunological responses of macrophages to PC@SMNs exposed to different pH values. a–d) ROS levels in PC@SMN‐treated dTHP‐1 cells quantified using microplate reader (MR) (a), FCM (b‐c), and CLSM (d). Lipopolysaccharide (LPS) was used as a positive control. e–g) Secretion of pro‐inflammatory cytokines: IL‐1*β* (g), TNF‐*α* (h), and IL‐6 (i) in dTHP‐1 cells treated with PC@SMNs for 12 h, quantified using ELISA. LPS or LPS + Nigericin serves as a positive controls. h) Schematic illustration of inflammatory responses of dTHP‐1 cell: PC@SMNs were taken up into lysosomes, inducing ROS production and activating inflammatory responses, which lead to the secretion of pro‐inflammatory cytokines (IL‐1*β*, TNF‐*α*, and IL‐6). Data are presented as mean ± standard deviation with *n* = 3 (biologically independent experiments). Statistical significance was tested with a two‐tailed, unpaired Student's *t*‐test, *p < 0.05, **p < 0.01, ***p < 0.001, and ****p < 0.0001 compared to the pH 7.4 group.

Recent studies have identified PC formation as a key mechanism by which nanomaterials trigger inflammatory responses.^[^ ^94^, ^95^, ^96^, ^97^, ^98^, ^99^ ^]^ Silica nanoparticles, in particular, are known to induce pro‐inflammatory cytokine production in immune cells.^[^ ^100^, ^101^, ^102^ ^]^ To investigate the inflammatory responses to PC@SMNs exposed to different pH conditions, macrophages (dTHP‐1) were incubated with PC@SMNs for 12 h. Elevated reactive oxygen species (ROS) levels are a hallmark of macrophage inflammatory responses induced by nanomaterials.^[^ ^103^ ^]^ ROS levels, measured using 2,7‐dichlorodihydrofluorescein diacetate (DCFH‐DA), showed a time‐dependent increase in dTHP‐1 cells (**Figure** **7**a). Notably, as the environmental pH decreased, ROS levels induced by PC@SMNs also decreased, as confirmed by FCM and CLSM analysis (Figure 7b–d). This suggests that while PC@SMNs exposed to lower pH conditions are more readily internalized by dTHP‐1 cells, they induce a weaker inflammatory response compared to those exposed to pH 7.4.

To further validate this phenomenon, inflammatory cytokines (IL‐1*β*, TNF‐*α*, and IL‐6) in the culture supernatant were measured using enzyme‐linked immunosorbent assay (ELISA) after treatment of dTHP‐1 cells with PC@SMNs. As shown in Figure 7e–g, cytokine levels significantly increased following PC@SMNs treatment, but decreased as the pH lowered, mirroring the trend observed in ROS levels. GO analysis suggests that this reduction may be due to the downregulation of immune‐related proteins in the PC exposed to pH 5.0 compared to those at pH 7.4 (Figure 3j).

The NF‐*κ*B signaling pathway, a key regulator of inflammatory cytokine expression, is known to be activated by nanomaterials (Figure 7 h)^.[^ ^104^, ^105^ ^]^ The reduced cytokine secretion and ROS levels under acidic conditions may result from decreased NF‐*κ*B activation, potentially caused by altered PC compositions. Several factors may contribute to this diminished inflammatory response: () lower abundance of complement protein C3 in the PC exposed to low pH may reduce nanoparticle recognition and opsonization, thus attenuating immune activation.^[^ ^46^, ^47^, ^106^ ^]^ 2) Elevated levels of C4BPA, a complement system regulatory protein, at low pH could enhance its inhibitory effect on complement pathway activation, further suppressing inflammatory responses and secretion of inflammatory cytokines.^[^ ^74^, ^75^ ^]^ 3) A decrease in immunoglobulins in the PC exposed to low pH may further diminish immune activation.^[^ ^99^ ^]^

## 3 Conclusion

In this study, we systematically investigated how physiological pH variations influence PC and, consequently, the cellular uptake and immunological responses of PC@SMNs. Our results reveal that environmental pH significantly impacts the hydrodynamic diameter, zeta potential, protein composition, and structural conformation of PC@SMNs, which in turn affects cellular interactions and biological outcomes. Protein adsorption on SMNs was shown to be highly pH‐sensitive, resulting in substantial shifts in the composition and structure of adsorbed proteins. In particular, lower pH conditions, such as those found in tumor microenvironments, increased the uptake of PC@SMNs by macrophages (RAW264.7 and dTHP‐1) and tumor cells (A549) due to reduced electrostatic repulsion and enhanced interactions with cell membranes. Despite the increased uptake, PC@SMNs exposed to lower pH induced a weaker inflammatory response of dTHP‐1 cells, as evidenced by reduced ROS levels and lower secretion of inflammatory cytokines (IL‐1*β*, TNF‐*α*, IL‐6). These attenuated inflammatory responses may stem from altered PC composition, characterized by the lower abundance of complement proteins (C3) and immunoglobulins, alongside an increase in the regulatory proteins such as C4BPA. This study highlights the critical role of environmental pH in modulating the biological behavior of NPs through changing their protein corona fingerprints, which is essential for the rational design of effective nanomedicines that can successfully navigate complex biological environments.

## 4 Experimental Section

### Chemicals

All chemicals used in the experiments were of analytical reagent grade and were utilized without further purification. Polyoxyethylene (5) nonylphenylether (Igepal CO‐520), oleic acid (OA, 96%), aqueous ammonia (NH_3_·H_2_O, 28%), tetraethyl orthosilicate (TEOS, 98%), (3‐aminopropyl) triethoxysilane (APTES, 99%), hydrochloric acid (HCl, ≈37%), sodium hydroxide (NaOH, 96%), ethanol (C_2_H_5_OH, 100%), iron (III) chloride hexahydrate (FeCl_3_·6H_2_O, 99%), hexane (97%), cyclohexane (99.5%), sodium dodecyl sulfate (SDS, 92.5‐100.5%), phorbol myristate acetate (PMA, 99.8%), and fluorescein isothiocyanate (FITC, 95%) were obtained from Aladdin Biochemistry Technology Co., Ltd (Shanghai, China). Sodium dodecyl sulfate‐polyacrylamide gel electrophoresis (SDS‐PAGE) precast gel, SDS‐PAGE rapid electrophoresis buffer for Hepes‐Tris system, SDS‐PAGE sample loading buffer (5x), and Coomassie blue superfast staining solution were purchased from Yeasen Biotechnology Co., Ltd (Shanghai, China). Phosphate‐buffered saline (PBS) solutions (pH 7.2–7.4, 0.01 M, cell culture), human serum albumin, (HSA, 96—99%), low‐density lipoprotein (LDL, 2 mg mL^−1^), and high‐density lipoprotein (HDL, 2 mg mL^−1^) were obtained from Solarbio Science & Technology Co., Ltd. (Beijing, China). Dulbecco's modified eagle medium (DMEM), Roswell Park Memorial Institute medium 1640 (RPMI‐1640), fetal bovine serum (FBS), and 0.25% ethylene diamine tetraacetic acid (EDTA)‐trypsin were purchased from Thermo Fisher Bio‐Chemicals Co., Ltd. (Beijing, China). BCA protein concentration assay kit, cell counting kit (CCK8), 4′, 6‐diamidino‐2‐phenylindole (DAPI), Hoechst 33342, Lyso‐Tracker Red, and 2,7‐dichlorodihydrofluorescein diacetate (DCFH‐DA) were provided by Beyotime Biotechnology Co., Ltd. (Shanghai, China). Chlorpromazine, methyl‐*β*‐cyclodextrin (M*β*CD), dynasore, 5‐(*N*‐ethyl‐*N*‐isopropyl) amiloride (EIPA), cytochalasin D, and nocodazole were purchased from MedChem Express BioTech Co., Ltd. (Monmouth Junction, NJ, USA). The IL‐1*β* ELISA kit, TNF‐*α* ELISA kit, and IL‐6 ELISA kit were obtained from Dakewe Biotech Co. Ltd (Shenzhen, China). Milli‐Q water was used in all experiments.

### Human Serum

Whole blood was obtained from ten apparently healthy blood donors, following the ethical guidelines outlined in the Declaration of Helsinki. Informed consent was obtained from all participants. The study was approved by the ethic committee at Ocean University of China (No.OUC‐HM‐2022‐01). The blood was stored in EDTA‐coated test tubes to prevent blood clotting. The blood samples were centrifuged at 1700 *g* for 5 min to precipitate erythrocytes and leukocytes. The resulting serum supernatant was mixed and stored at −80 °C.

### Preparation of PBS Solutions with Different pH Values

To prepare PBS solutions with pH values of 7.4, 7.0, 6.5, 6.0, 5.5, 5.0, and 4.5, the pH values of PBS (pH 7.2‐7.4, 0.01 M) were adjusted using 0.1 M HCl and 0.1 M NaOH aqueous solutions. The pH values of PBS solutions were measured using a pH electrode (Mettler Toledo, FE28‐standard).

### Synthesis of Fe3O4 Nanoparticles

Fe_3_O_4_ NPs were synthesized following a procedure outlined in the literature.^[^ ^36^ ^]^ In a typical synthesis, FeCl_3_·6H_2_O (2 mmol) was dissolved in H_2_O (6 mL). Ethanol (8 mL), hexane (14 mL), and oleic acid (1.9 mL) were sequentially added. After stirring at room temperature for 30 min, NaOH (0.24 g) was added, and the mixture was reacted in a sealed vessel at 70 °C for 4 h. The resulting solution was separated into two layers using a separatory funnel. The organic layer containing the Fe(oleate)_3_ complex was collected, washed three times with deionized water, and then dried overnight at 80 °C to remove hexane. The viscous Fe(oleate)_3_ precursor was dispersed in OA (0.32 mL) and 1‐octadecene (12.5 mL). N_2_ purging was performed for 30 min, followed by heating under N_2_ atmosphere at 320 °C for 30 min. After cooling to room temperature, the nanoparticles were precipitated by adding ethanol (50 mL), collected by centrifugation, and the supernatant was decanted. The solid was redispersed in hexane and precipitated with ethanol. This precipitation‐redispersion process was repeated three times to purify the Fe_3_O_4_ NPs.

### Synthesis of Silica‐Coated Magnetic Nanoparticles (SMNs)

SMNs were prepared using a reverse microemulsion method.^[^ ^36^ ^]^ Specifically, Igepal CO‐520 (0.5 g) was dispersed in cyclohexane (11 mL) and sonicated for 10 min. Then, Fe_3_O_4_ nanoparticles (4 mL, 1 mg mL^−1^ in cyclohexane, 12.2 nm diameter) were added under stirring. Subsequently, NH_3_·H_2_O (0.1 mL) was added to the mixture, followed by dropwise addition of TEOS (0.1 mL, 25 *µL* every 12 h). The FITC‐labelled SMNs were prepared by adding an additional FITC‐APTES (25 *µ*L). The FITC‐APTES was prepared at a 1:15 molar ratio of FITC to APTES, with FITC (2 mg) dissolved in methanol (2 mL), to which 18.1 *µ*L of APTES was added. The reaction proceeded under light‐protected conditions with vigorous stirring (25 °C, 1000 rpm, 48 h). SMNs were harvested by centrifugation (11100 *g*, 20 min), washed five times with anhydrous ethanol (15 mL per wash). The final washed precipitate was stored in PBS (0.01 M, pH 7.4) buffer or ultrapure water for further experiments.

### Characterization of SMNs

The morphology of SMNs was characterized using transmission electron microscopy (TEM, JEOL, JEM‐1200EX). The hydrodynamic diameter (*D*_h_) and zeta potential of SMNs were determined using a Zetasizer Nano ZS (Malvern, ZEN3600). The results are calculated from an average of three consecutive measurements.

### Preparation of PC@SMNs

Method 1 mimics static exposures to a series of pathophysiologically relevant pH values across various disease contexts (Figure 2a), while Method 2 models stepwise and progressive acidification that nanoparticles undergo during tumor‐targeted delivery (Scheme S1, Supporting Information). Method 1: To prepare PC@SMNs, SMNs (500 *µ*L, 2 mg mL^−1^, in pH 7.4 PBS) were mixed with an equal volume of human serum and incubated at 37 °C for 1 h. The mixture was then centrifuged at 17970 *g* for 30 min at 4 °C to remove unbound proteins. Subsequently, PBS buffer solutions (1 mL, 0.01 M) with different pH values (pH 7.4, 7.0, 6.5, 6.0, 5.5, 5.0, or 4.5) were added and incubated again at 37 °C for 1 h. The final mixture was centrifuged at 17970 *g* for 30 min at 4 °C to obtain PC@SMNs complexes. The PC@SMNs prepared using the aforementioned steps were used for DLS characterization, BCA, and SDS‐PAGE experiments.

Method 2: To simulate the pH transitions encountered by PC@SMNs during in vivo tumor delivery, SMNs (500 *µ*L, 2 mg mL^−1^, in pH 7.4 PBS) were mixed with an equal volume of human serum and incubated at 37 °C for 1 h. The mixture was then centrifuged at 17970 *g* for 30 min at 4 °C to remove unbound proteins. Subsequently, PBS buffer (1 mL, pH 7.4, 0.01 M) was added to the precipitation, and the suspension was incubated at 37 °C for 1 h again. The resulting mixture was centrifuged at 17970 *g* for 30 min at 4 °C to obtain PC@SMNs (pH 7.4). To prepare PC@SMNs (pH 6.5), the pH values of the solution were further adjusted to 6.5 using HCl (0.01 M). The mixture was incubated at 37 °C for another 1 h to mimic the mildly acidic environment within tumor tissues. The final mixture was centrifuged under the same conditions to yield PC@SMNs (pH 6.5). To obtain PC@SMNs (pH 5.0), the pH values of the solution were further adjusted to 5.0 using HCl (0.01 M). This step aimed to simulate the acidic environment encountered within lysosomes. The mixture was incubated at 37 °C for 1 h and centrifuged at 17970 *g* for 30 min at 4 °C to produce PC@SMNs (pH 5.0). The PC@SMNs prepared using the aforementioned steps were used for LC‐MS/MS and cellular experiments.

### Characterization of PC@SMNs

*D*_h_ and zeta potential of PC@SMNs exposed to different pH values (pH 7.4, 7.0, 6.5, 6.0, 5.5, 5.0, or 4.5) was measured using a Zetasizer Nano ZS (Malvern, ZEN3600). All measurements were performed in PBS buffer (0.01 M) at the corresponding pH values, and each sample was measured at least three times to ensure data reliability.

### Protein Quantification by Using BCA Assay

Protein adsorption on SMNs was quantified using a Pierce BCA protein assay. PC@SMNs complexes were incubated with SDS‐Tris buffer (100 *µL*, 2% SDS, 62.5 mM Tris‐HCl) at 95 °C for 5 min to desorb bound proteins. The detached proteins were separated from SMNs by centrifugation (17970 *g* for 1 h at 4 °C). The protein concentration was quantified using the BCA protein assay kit. Protein sample solution (10 *µL*) was added to the working reagent (200 *µL*) and incubated at 37 °C for 30 min. The absorbance at 562 nm was detected using a microplate reader (MR, Tecan, Spark).

### Protein Separation by Using SDS‐PAGE

The presence of proteins on the surface of SMNs was verified by SDS‐PAGE. Desorbed protein solutions were mixed with protein loading buffer, heated at 95 °C for 5 min. After cooling, the prepared samples were loaded onto a 4–20% SDS‐PAGE gel and electrophoresed at a constant voltage (150 V, 45 min) until the lowest molecular weight proteins approached the bottom of the gel. The gel was then stained with Coomassie Brilliant Blue to visualize the protein bands. Grayscale analysis of the bands was performed using Image J software (“Analyze→Gels→Plot Lanes” function) for quantification.

### Protein Fingerprinting Analysis by Using LC‐MS/MS

The desorbed protein solution was diluted 10‐fold with a 10% (w/v) SDS aqueous solution. The mixture was incubated at 95 °C for 5 min and then centrifuged (17970 *g*, 1 h, 4 °C). Protein in the supernatant was precipitated with trichloroacetic acid (TCA), and the precipitation was resuspended in redissolved solution (8 M urea, 100 mM Tris ‐HCl, pH 8.5). Protein concentration was quantified using BCA assay. Equal amounts (20 µg per sample) were used for the subsequent steps. Proteins were reduced with tris(2‐carboxyethyl)phosphine (TCEP) and alkylated with chloroacetamide (CAA) at 37 °C for 1 h. The urea concentration was diluted to <2 M using Tris‐HCl (100 mM). Trypsin (1:50, enzyme:protein, w:w) was added for overnight digestion at 37 °C. The next day, digestion was terminated by adjusting pH to 6.0 with trifluoroacetic acid (TFA). After centrifugation (12000 *g*, 15 min), peptides were desalted using a self‐made SDB‐RPS desalting column. Purified peptides were lyophilized and stored at −20 °C for later use.

### Mass Spectrometric Detection

All samples were analyzed using an UltiMate 3000 RSLCnano system (Thermo Fisher Scientific, USA) coupled with a Q Exactive HF mass spectrometer, incorporating a Nanospray Flex ion source. Peptide samples were injected into a C18 Trap column (75 *µ*m × 2 cm, 3 *µ*m particle size, 100 Å pore size), and separated in a reversed‐phase C18 analytical column packed with ReproSil‐Pur C18‐AQ resin (75 *µ*m × 25 cm, 1.9 *µ*m particle size, 100 Å pore size). A binary solvent system was employed for elution, consisting of mobile phase A (0.1% formic acid, 3% DMSO, 97% H_2_O) and mobile phase B (0.1% formic acid, 3% DMSO, 97% acetonitrile). A gradient elution was applied at a flow rate of 300 nL min^−1^. The mass spectrometer was configured in data‐dependent acquisition (DDA) mode, selecting the top 20 most abundant ions for fragmentation. Full mass spectra were acquired over a range of 350–1500 mz^−1^ with an AGC target of 3E6 charges. The injection time was capped at 30 ms, and a resolution of 60000 at mz^−1^ 200 was applied. For precursor ion selection, a window of 1.4 mz^−1^ was maintained. Fragmentation was carried out using higher‐energy collisional dissociation (HCD) with a normalized collision energy of 28. Fragment ion scans were recorded at a resolution of 15000 with an AGC of 1E5, and a maximum fill time of 50 ms. A dynamic exclusion period was set to 30 s to avoid repetitive sampling of abundant ions.

### Database Search

The raw mass spectrometry data were processed using MaxQuant (V1.6.6.0) software, which employs the Andromeda database search algorithm. Spectra files were matched against the Human protein sequence database (2023‐06‐19, 20 423 entries). Protein sequences retrieved from Uniprot were analyzed using the following parameters: oxidation (M), acetyl (Protein N‐term), and deamidation (NQ) as variable modifications, while Carbamidomethyl (C) was assigned as a fixed modification. Trypsin/P was selected as the digestion enzyme, allowing up to two missed cleavages. The mass tolerance for the first search and main search was set at 20 ppm and 4.5 ppm, respectively. Proteins sharing unique peptides were grouped together by MaxQuant, and results were filtered at 1% false discovery rate (FDR) at both peptide and protein levels.

### Bioinformatic Analysis

Heat maps displaying normalized protein abundance were generated via an online platform (https://www.bioinformatics.com.cn). The function of PC composition was categorized using the DAVID website (https://david.ncifcrf.gov/). Proteins with significantly altered abundance were discriminated based on a fold change of ≥ 1.5 and *P* < 0.05, with volcano plots used for visualization. To analyze biological functions, Gene Ontology (GO) and Kyoto Encyclopedia of Genes and Genomes (KEGG) pathways were performed using the “clusterProfiler” R package. The net plots were used to illustrate functional enrichment and biological process terms.

### Fluorescence Quenching Studies

HSA solutions (500 µg mL^−1^) were prepared in PBS buffers at various pH values (0.01 M, pH 7.4, 7.0, 6.5, 6.0, 5.5, 5.0, or 4.5). For fluorescence quenching studies, after incubating at 37 °C for 1 h, the fluorescence spectra were recorded using a fluorescence spectrometer (Horiba, FluoroMax‐4). Heat‐denatured HSA (100 °C, 10 min) served as a positive control, and a PBS buffer (0.01 M, pH 7.4) was used as a negative control. For interaction studies, HSA solutions (1 mg mL^−1^) with different pH levels were mixed with equal volumes of SMN solutions at 10, 20, 50, 100, 200, or 500 µg mL^−1^. After incubating at 37 °C for 1 h, the changes in HSA fluorescence spectra were measured. The excitation wavelength was set to 280 nm with a slit width of 3 nm. SMN solutions at the same concentrations were used as controls.

### UV–Vis Spectroscopy

HSA solutions (500 µg mL^−1^) were prepared in PBS buffers at various pH values (0.01 M, pH 7.4, 7.0, 6.5, 6.0, 5.5, 5.0, or 4.5). After incubating at 37 °C for 1 h, the UV–vis spectra were recorded using a UV‐vis spectrophotometer (Shimadzu, UV‐1800). Heat‐denatured HSA (100 °C, 10 min) served as a positive control and PBS buffer (0.01 M, pH 7.4) was used as negative control. For interaction studies, HSA solutions (1 mg mL^−1^) with different pH levels were mixed with equal volumes of SMN solutions (200 µg mL^−1^). After incubating at 37 °C for 1 h, the UV–vis spectra of HSA were measured using a UV–vis spectrophotometer (Shimadzu, UV‐1800), with spectra ranging from 200 to 500 nm. SMN solution (100 µg mL^−1^) was used as the reference solution.

### CD Spectroscopy

HSA solutions (250 µg mL^−1^) were prepared in PBS buffers at various pH values (0.01 M, pH 7.4, 7.0, 6.5, 6.0, 5.5, 5.0, or 4.5). After incubating at 37 °C for 1 h, CD spectra were measured using a CD spectropolarimeter (Jasco, J‐815) in a quartz cuvette (path length = 0.1 cm, scanning range = 190–260 nm). Measurements were conducted three times (digital integration time = 2 s, bandwidth = 1 nm, data interval = 0.5 nm, scanning speed = 100 nm min^−1^). The PBS buffer (0.01 M, pH 7.4) was used for baseline correction. Heat‐denatured HSA (250 µg mL^−1^, 100 °C for 10 min) served as a positive control. The CD spectra were analyzed for secondary structure content using the CDSSTR algorithm in CDPro software. For interaction studies, HSA solutions (500 µg mL^−1^) with different pH levels were mixed with equal volumes of SMN solutions (100 µg mL^−1^). After incubating at 37 °C for 1 h, the CD spectra of HSA were measured under the same conditions as described above. SMN solution (50 µg mL^−1^) served as a negative control. All experiments were conducted under N_2_ (0.4 MPa).

### Cell Culture

Mouse mononuclear macrophage cells (RAW264.7), human non‐small cell lung cancer cells (A549), and human monocytic leukemia cells (THP‐1) were purchased from Type Culture Collection of Chinese Academy of Sciences (Shanghai, China). All cells were cultured in RPMI‐1640 or DMEM supplemented with 10% FBS and 1% penicillin/streptomycin at 37 °C in a CO_2_ incubator (95% relative humidity, 5% CO_2_). Macrophage‐like THP‐1 (dTHP‐1) cells were differentiated from THP‐1 cells by treatment with PMA (100 ng mL^−1^, 48 h).

### Cell Viability

Cell viability was assessed using the CCK‐8 assay in RAW264.7, A549, and THP‐1 cells seeded at 1 × 10^4^ cells/well in 96‐well plates. After 24 h of culture, cells were treated with various concentrations of SMNs (15.625, 31.25, 62.5 125, 250, or 500 µg mL^−1^) for 24 h. For THP‐1 cells, they were differentiated into macrophages with PMA (100 ng mL^−1^) at 37 °C for 48 h, followed by a further 24 h culture before SMNs treatment. Cell viability was determined by adding CCK‐8 reagent (10 *µL*) and measuring absorbance at 480 nm after 1–4 h, as per the optimization results. Experiments were repeated three times.

### Cellular Uptake

RAW264.7, A549, and THP‐1 cells were cultured in six‐well plates at 5 × 10^5^ cells/well (RAW264.7, A549) or 1 × 10^6^ cells/well (THP‐1) and treated with PC@SMNs (200 µg mL^−1^) in serum‐free cell culture medium for 1, 2, 4, 6, and 8 h. SMNs were labelled with FITC. THP‐1 cells were differentiated into macrophages with PMA (100 ng mL^−1^) at 37 °C for 48 h, followed by a culture for 24 h before treatment with PC@SMNs. After exposure, cells were washed three times with PBS, digested with trypsin, and centrifuged at 130 *g* for 3 min. The collected cells were analyzed by FCM (Beckman, FC500), with each sample measured three times. For CLSM, cells were seeded in 20 mm confocal culture dishes at 2 × 10^5^ cells/well. After exposure, cells were washed three times with PBS and fixed with 4% paraformaldehyde for 20 min. Cells were washed three times with PBS, then stained with DAPI for 10 min to label the nuclei. After staining, cells were washed three times with PBS (0.01 M, pH 7.4), and PBS (1 mL, 0.01 M, pH 7.4) was added to each dish. Stained cells were observed using the CLSM (Leica, TCS SP8) with a 63x oil immersion objective at excitation wavelengths of 405 and 488 nm. Semi‐quantitative analysis of FITC fluorescence intensity was performed using ImageJ software.

### Cellular Uptake and Protein Abundance Correlation Analysis

To investigate the relationship between cellular uptake of PC@SMNs exposed to different pH conditions and the composition of the protein corona, Pearson correlation analysis was performed to evaluate the correlation between fluorescence intensity of cellular uptake and protein abundance by GraphPad Prism 9.0. The results were visualized using a volcano plot.

### Cellular Uptake with Endocytosis Inhibitor or Competitive Blockers

RAW264.7, A549, and THP‐1 cells were cultured in 6‐well plates at 5 × 10^5^ cells/well (RAW264.7, A549) or 1 × 10^6^ cells/well (THP‐1). Cells were pre‐treated with endocytosis inhibitors or competitive blockers for 1 h before exposure to PC@SMNs, followed by FCM (Beckman, FC500) analysis, with each sample measured three times. For THP‐1 cells, they were differentiated into macrophages with PMA (100 ng mL^−1^) at 37 °C for 48 h, followed by a further 24 h culture before treatment. The concentrations of the various endocytosis inhibitors and competitive blockers were as follows: for RAW264.7 cells, chlorpromazine (5 µg mL^−1^), M*β*CD (2.5 mg mL^−1^), dynasore (100 *µ*M), EIPA (100 *µ*M), cytochalasin D (2.5 µg mL^−1^), nocodazole (5 *µ*M), fucoidan (2 mg mL^−1^), LDL (25 µg mL^−1^), and HDL (25 µg mL^−1^); for dTHP‐1 cells, chlorpromazine (10 µg mL^−1^), M*β*CD (2.5 mg mL^−1^), dynasore (100 *µ*M), EIPA (100 *µ*M), cytochalasin D (2.5 µg mL^−1^), nocodazole (5 *µ*M), fucoidan (2 mg mL^−1^), LDL (25 µg mL^−1^), and HDL (25 µg mL^−1^); for A549 cells, chlorpromazine (10 µg mL^−1^), M*β*CD (2.5 mg mL^−1^), dynasore (50 *µ*M), EIPA (50 *µ*M), cytochalasin D (2.5 µg mL^−1^), nocodazole (5 *µ*M), fucoidan (2 mg mL^−1^), LDL (25 µg mL^−1^), and HDL (25 µg mL^−1^).

### Intracellular Localization of PC@SMNs by Using CLSM

RAW264.7, A549, and THP‐1 cells were cultured in 20 mm confocal culture dishes at 2 × 10^5^ cells/well and treated with PC@SMNs (200 µg mL^−1^) for 2 h, 4 h, and 6 h. SMNs were labelled with FITC. For THP‐1 cells, they were differentiated into macrophages with PMA (100 ng mL^−1^) at 37 °C for 48 h, followed by a further 24 h culture before PC@SMNs treatment. After exposure, cells were washed three times with PBS to remove unbound PC@SMNs. Then, cells were stained with Lyso‐Tracker Red for lysosomes and Hoechst 33 342 for nuclei, according to the manufacturer's instructions. After staining, cells were washed three times with PBS to remove excess dye. Then, PBS (1 mL, 0.01 M, pH 7.4) was added to each dish to keep the cells hydrated. Cells were observed using the CLSM (Leica, TCS SP8). Hoechst 33 342 was excited with a 405 nm laser (blue), and Lyso‐Tracker Red was excited with a 561 nm laser (red). Images were acquired, and colocalization analysis was performed to determine the intracellular localization of PC@SMNs using ImageJ software.

### Measurement of ROS in Macrophages by Using MR, FCM, and CLSM

The effect of PC@SMNs on ROS production in dTHP‐1 cells was assessed using the DCFH‐DA assay. THP‐1 cells were seeded in 96‐well plates at 1 × 10^4^ cells/well and were differentiated into macrophages with 100 ng mL^−1^ PMA at 37 °C for 48 h, followed by a further 24 h culture. For MR, the medium was replaced with DCFH‐DA/1640 medium (100 *µL*, final concentration of DCFH‐DA: 10 *µ*M) and incubated at 37 °C for 20 min. Cells were exposed to PC@SMNs (200 µg mL^−1^), and the fluorescence intensity of DCF was measured at different time points using the MR (Tecan, Spark) at 488 nm excitation and 525 nm emission. The fluorescence intensity was used to evaluate ROS levels in dTHP‐1 cells. For FCM and CLSM, cells were exposed to PC@SMNs (200 µg mL^−1^) for 12 h, trypsinized, and centrifuged at 130 *g* for 3 min. Cells were resuspended in DCFH‐DA/1640 medium (final concentration of DCFH‐DA: 10 *µ*M) and incubated at 37 °C for 20 min. Cells were washed three times with PBS to remove excess dye. Fluorescence intensity at 488 nm was measured using the FCM (Beckman, FC500). The fluorescence intensity was used to evaluate ROS levels in dTHP‐1 cells. For CLSM, cells were observed using the CLSM (Leica, TCS SP8). Images were acquired and quantitatively analyzed for ROS levels using ImageJ software.

### Detection of Cytokine Release by ELISA

THP‐1 cells were seeded in 96‐well plates at 1 × 10^4^ cells/well and were differentiated into macrophages with PMA (100 ng mL^−1^) at 37 °C for 48 h, followed by a further 24 h culture. Next, dTHP‐1 cells were stimulated with LPS (100 ng mL^−1^) for 3 h and treated with PC@SMNs in serum‐free medium for 12 h. Culture supernatants were collected, and the levels of IL‐1*β*, TNF‐*α*, and IL‐6 were detected using ELISA kits. Specifically, 96‐well plates were coated with purified anti‐human capture monoclonal antibodies according to the kit instructions. 100 *µL*/well culture supernatants or protein standards were added to each well and incubated at 37 °C for 3 h. After incubation, wells were washed three times with 300 *µL*/well washing buffer. 50 *µL*/well biotinylated anti‐human antibodies were added and incubated at 37 °C for 1 h. Wells were washed three times again. 100 *µL*/well avidin‐horseradish peroxidase (HRP) solution was added and incubated at 37 °C for 30 min. Wells were washed three times after incubation. 100 *µL*/well substrate solution was added and incubated at 37 °C for 15 min in the dark. The reaction was stopped by adding 100 *µL*/well stop solution. Absorbance was measured at 450 nm using the MR (Tecan, Spark). Experiments were performed in triplicate, and results were expressed in pg mL^−1^. Cytokine concentrations in the samples were calculated using standard curves.

### Statistical Analysis

Statistical significance was assessed using Student's *t*‐test or one‐way ANOVA, as appropriate, in GraphPad Prism 9.0. *p* < 0.05 was considered statistically significant compared to the control group or pH 7.4 group. Statistical differences were indicated as follows: *p < 0.05, **p < 0.01, and ***p < 0.001, representing significant, more significant, and the most significant differences, respectively.

## Conflict of Interest

The authors declare no conflict of interest.

## Author Contributions

Y. G. and F. F. contributed equally to this work. Supervision is performed by S.J. and K.L. Conceptualization is performed by Y.T.G., F.Q.F., V.M., D.C, S.J., and K.L. Methodology is done by Y.T.G., F.Q.F., Y.G., and T.C.H. Investigation is performed by Y.T.G., F.Q.F., Y.G., and S.J. Visualization is done by Y.T.G., F.Q.F., and Y.G. Writing (original draft) is done by Y.T.G and S.J. Writing (review and editing) is done by S.J, V.M., D.C, and K.L. All authors discussed the results and commented on the paper.

## Supporting information



Supporting Information

## Data Availability

The data that support the findings of this study are available from the corresponding author upon reasonable request.
