# Supplementary material for: Physiological pH Transition‐Driven Protein Corona Dynamics Regulate Cellular Uptake and Inflammatory Responses of Silica Nanoparticles
Source: Adv Sci (Weinh). 2025 Sep 3;12(43):e02788. doi: 10.1002/advs.202502788 (PMC12631826; doi:10.1002/advs.202502788)
Supplement: Supplementary file 1 — Supporting Information [file ADVS-12-e02788-s001.docx]

# Supporting Information

**Physiological pH Transition-Driven Protein Corona Dynamics Regulate Cellular Uptake and Inflammatory Responses of Silica Nanoparticles**

*Yuting Ge,^+^ Fangqin Fu,^+^ Yu Gao, Tianchang He, Volker* *Mailänder, Daniel Crespy*_,_ *Katharina Landfester,* Shuai Jiang**

Y. Ge, F. Fu, Y. Gao, T. He, S. Jiang

Key Laboratory of Marine Drugs, Chinese Ministry of Education, School of Medicine and Pharmacy, Ocean University of China, Qingdao 266003, P. R. China

E-mail: [jiangshuai@ouc.edu.cn](mailto:jiangshuai@ouc.edu.cn)

Y. Ge, F. Fu, Y. Gao, T. He, S. Jiang

Laboratory for Marine Drugs and Bioproducts, Qingdao Marine Science and Technology Center, Qingdao, 266237, P. R. China

V. Mailänder, K. Landfester

Max Planck Institute for Polymer Research, Ackermannweg 10, 55128 Mainz, Germany

E-mail: [landfester@mpip-mainz.mpg.de](mailto:landfester@mpip-mainz.mpg.de)

D. Crespy

Department of Materials Science and Engineering, School of Molecular Science and Engineering, Vidyasirimedhi Institute of Science and Technology (VISTEC), Rayong 21210, Thailand

[+] These authors contributed equally to this work.

# Supplementary Scheme


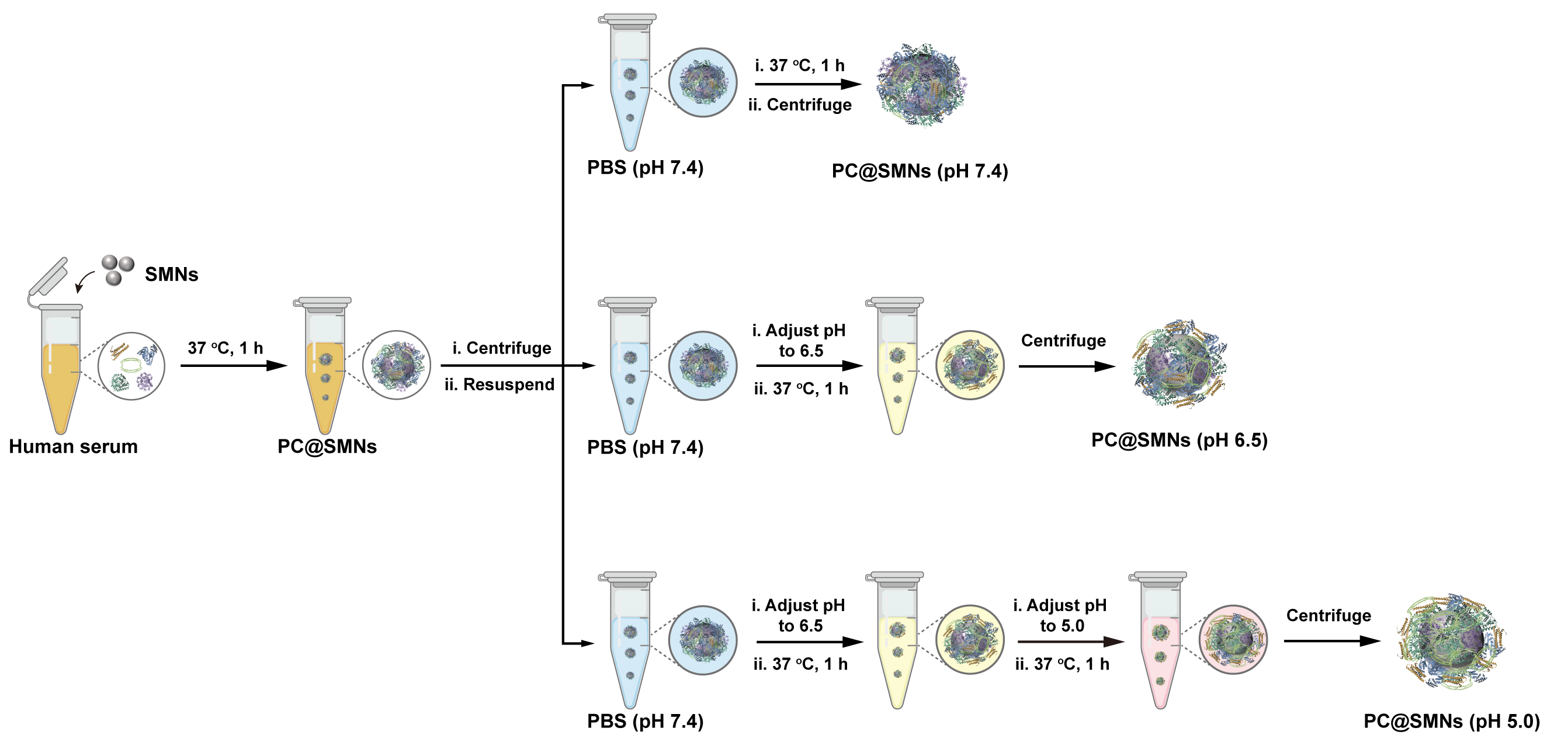


Scheme S1. Schematic illustration of the stepwise preparation of PC@SMNs exposed to physiologically relevant pH transitions. SMNs were first incubated with human serum at 37 °C to allow protein corona formation. Unbound proteins were removed by ultracentrifugation to obtain the preformed PC@SMNs (Pre-PC@SMNs). Next, the Pre-PC@SMNs were incubated again in PBS (pH 7.4) to simulate blood circulation. To mimic the acidification of TME, the pH of incubated PC@SMNs (pH 7.4) was then adjusted to 6.5 and incubated again. To simulate lysosomal conditions, the pH of the sample was further adjusted from 6.5 to 5.0 and incubated again. After each step, PC@SMNs (pH 7.4, 6.5, or 5.0) were collected by centrifugation.

# Supplementary Figures


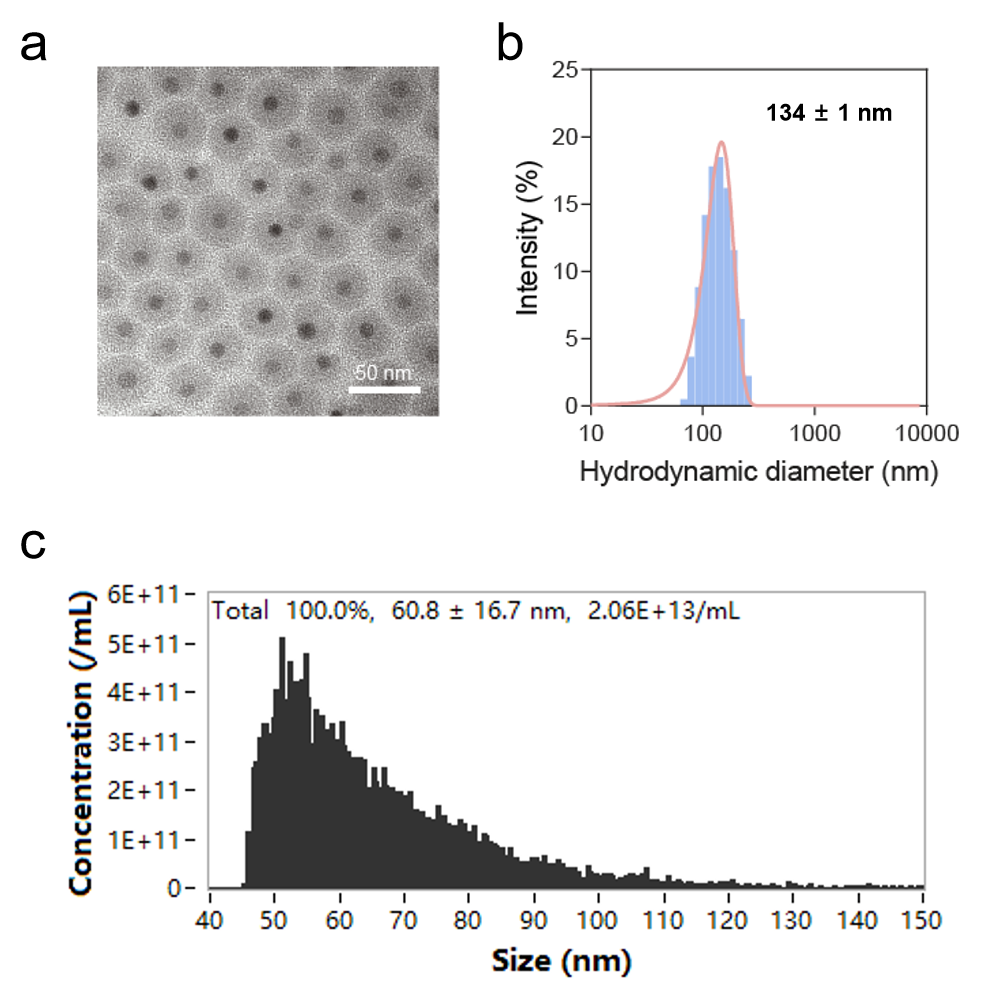


**Figure S1. Characterization of SMNs.** **(a)** TEM image of SMNs. **(b)** Intensity-weighted size distribution of bare SMNs in PBS (pH 7.4) measured by using DLS. (c) Size distribution of bare SMNs in PBS (pH 7.4) measured by using nanoFCM.


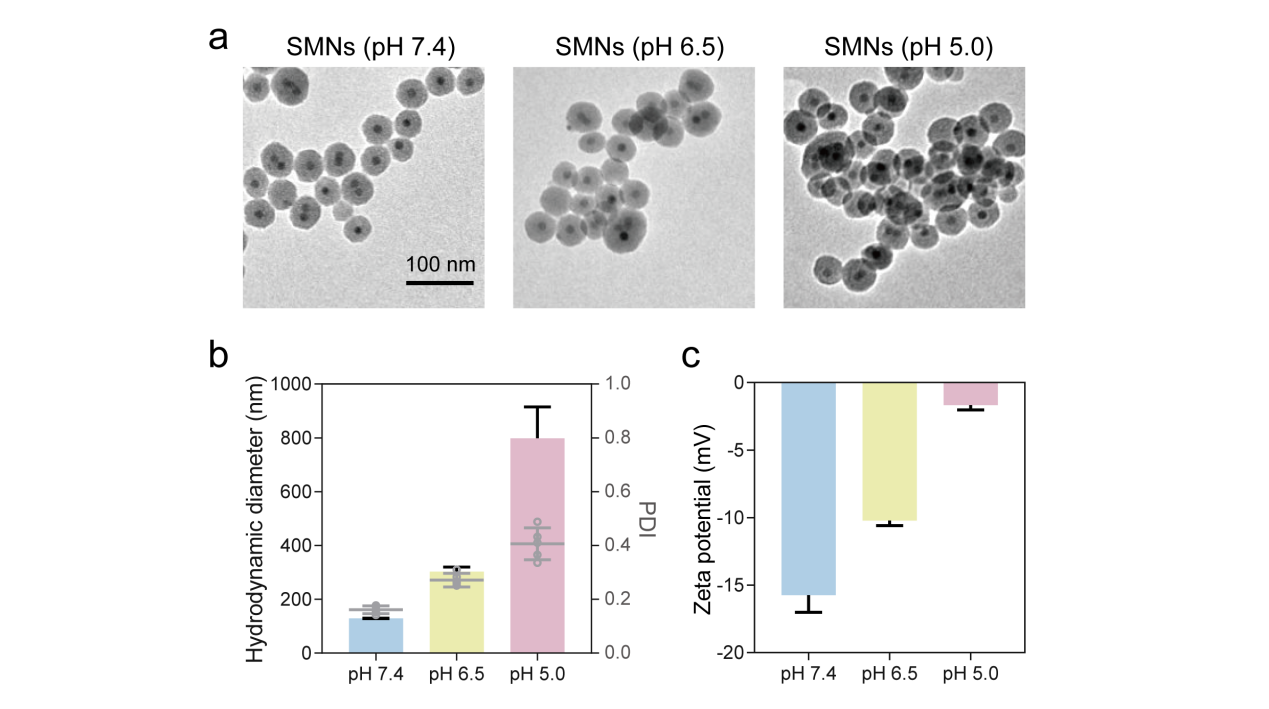


**Figure S2**. TEM images **(a)**, hydrodynamic diameter **(b)**, and zeta potential **(c)** of bare SMNs dispersed in PBS (pH 7.4, 6.5, or 5.0). Scale bar: 100 nm.


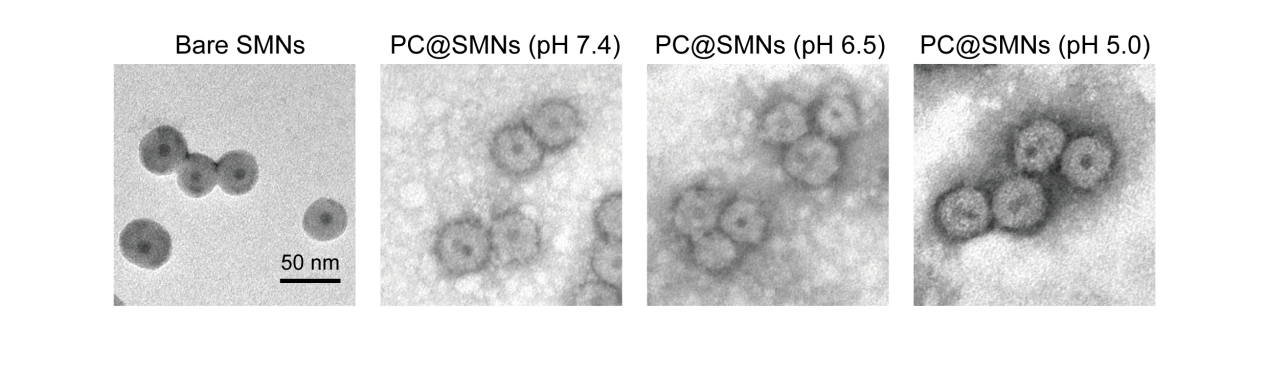


**Figure S3.** TEM images of bare SMNs and PC@SMNs exposed to PBS with pH 7.4, 6.5, or 5.0. Scale bar: 50 nm.


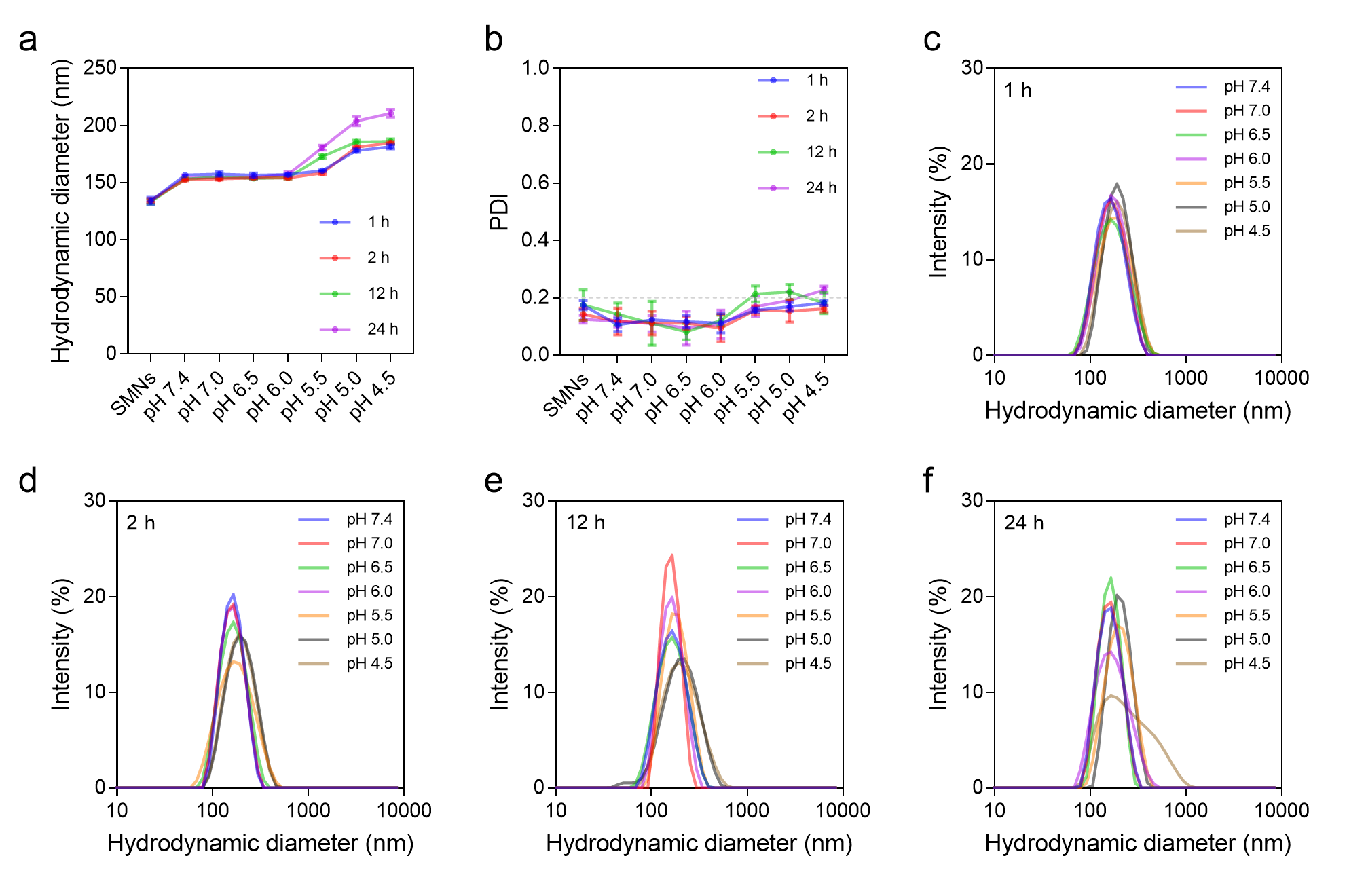


**Figure S4. Characterization of the hydrodynamic diameter of PC@SMNs at different time points using DLS**. Hydrodynamic diameter **(a)** and PDI **(b)** of PC@SMNs incubated at pH 7.4~4.5 for 1 h, 2 h, 12 h, and 24 h. Size distribution profiles of PC@SMNs at 1 h **(c)**, 2 h **(d)**, 12 h **(e)**, and 24 h **(f)**. Data are presented as mean ± SD (*n* = 3 independent measurements).


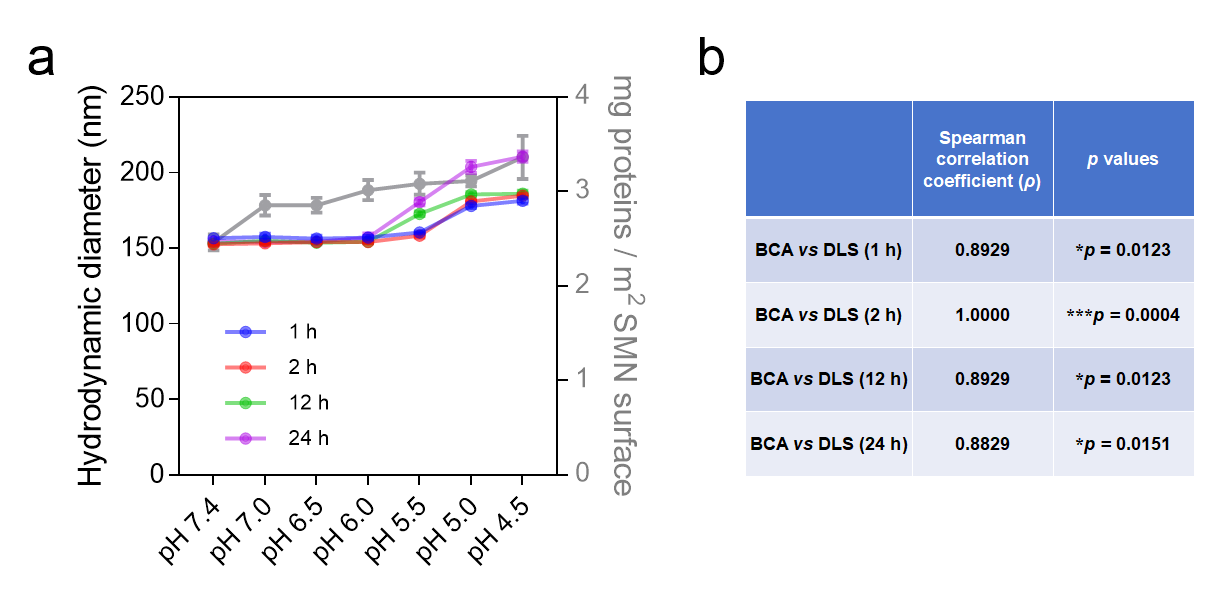


**Figure S5. (a)** Comparison of protein adsorption (right y-axis) and hydrodynamic diameter (left y-axis) at different time points (1 h, 2 h, 12 h, and 24 h). **(b)** Spearman correlation analysis between BCA results and DLS-measured hydrodynamic diameter at each time point. Data are presented as mean ± SD (*n* = 3, independent measurements).

**
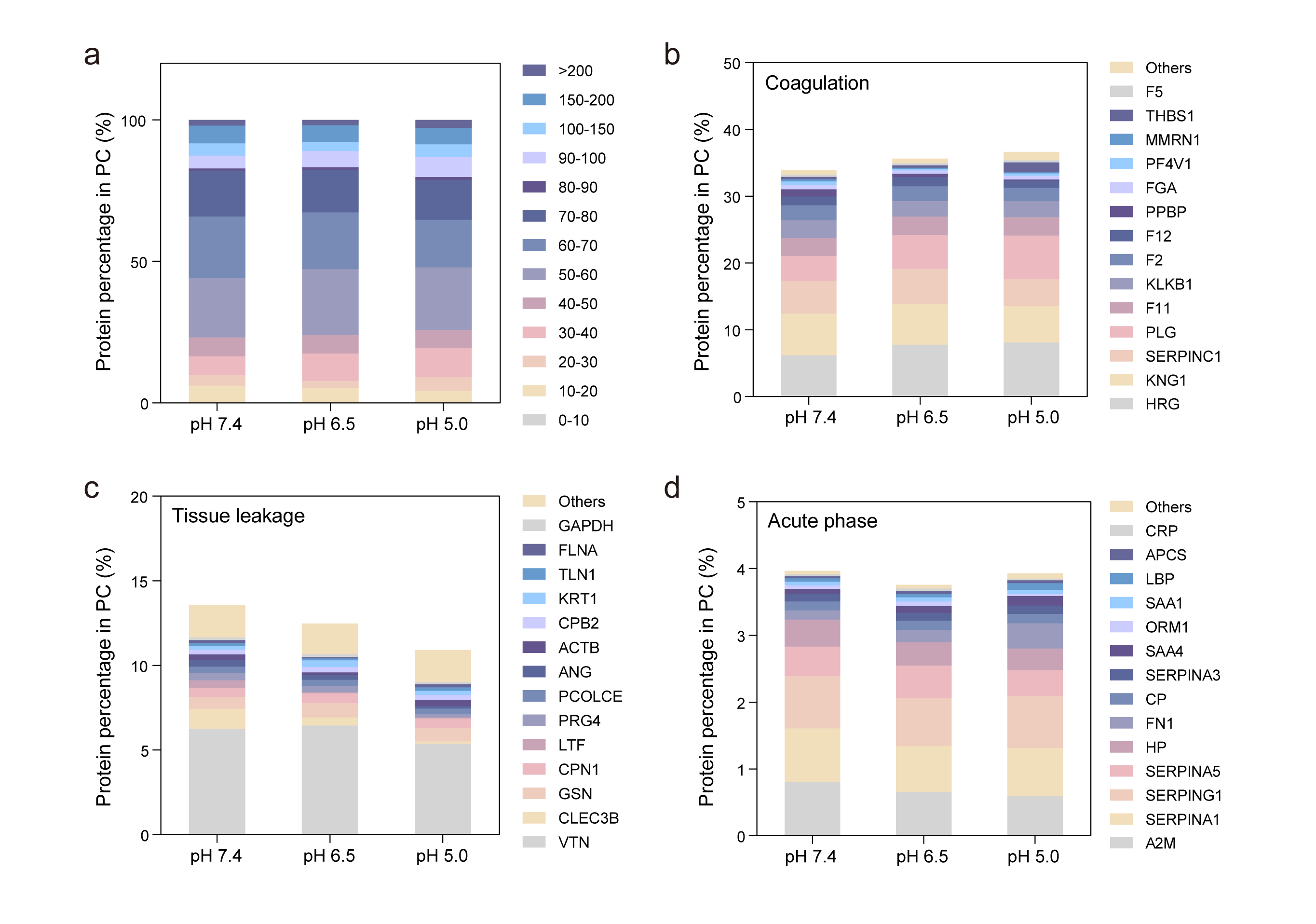
**

**Figure S6. (a)** Classification of proteins identified in PC@SMNs according to their calculated molecular weight. **(b-d)** Relative abundance of the identified coagulations **(b)**, tissue leakage proteins **(c)**, and acute phase proteins **(d)** in PC@SMNs.

**
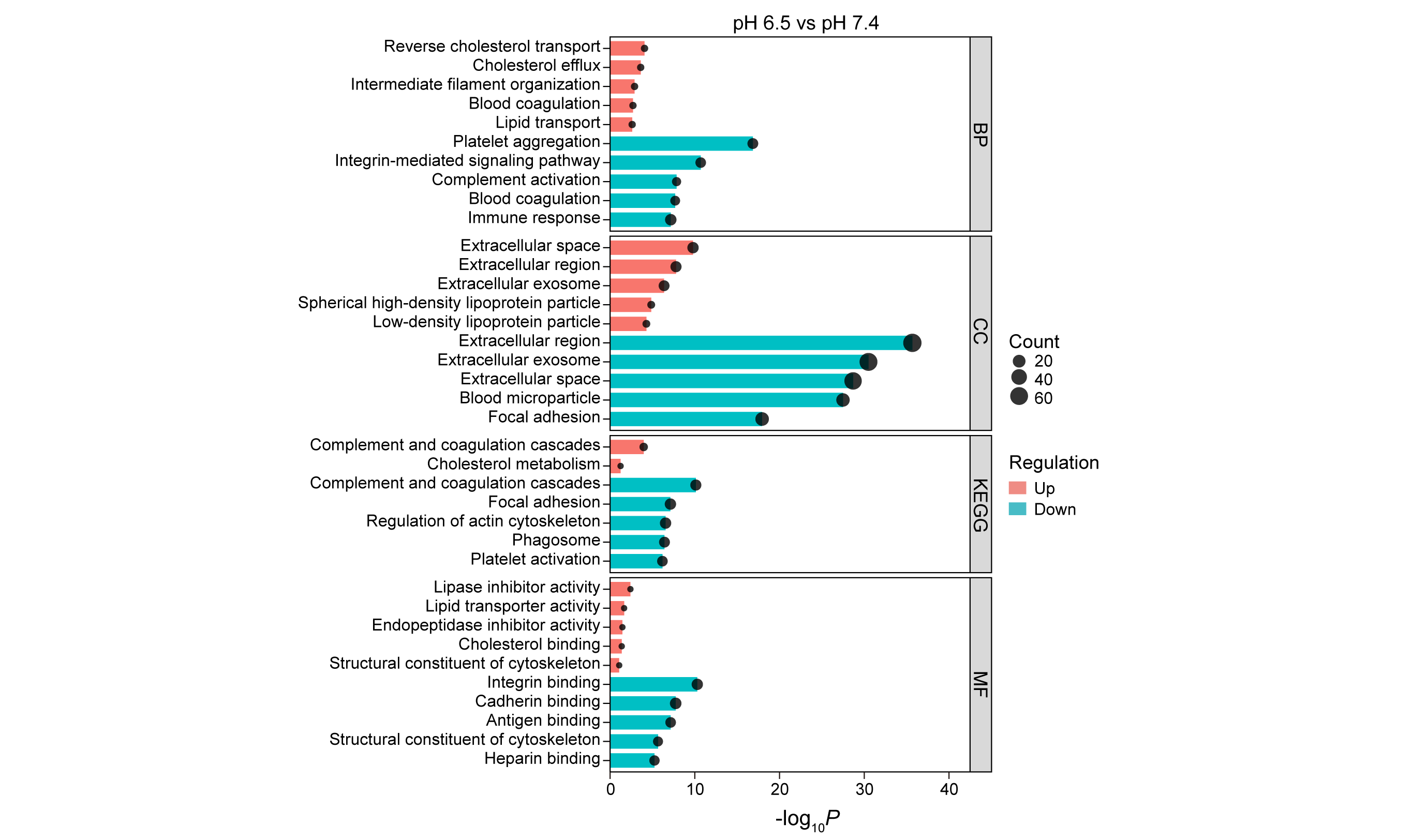
**

**Figure S7.** **GO and KEGG analyses of significantly upregulated and downregulated proteins in PC@SMNs (pH 6.5) compared to PC@SMNs (pH 7.4).** GO, gene ontology; BP, biological process; CC, cell components; MF, molecular functions; KEGG, Kyoto Encyclopedia of Genes and Genomes.

**
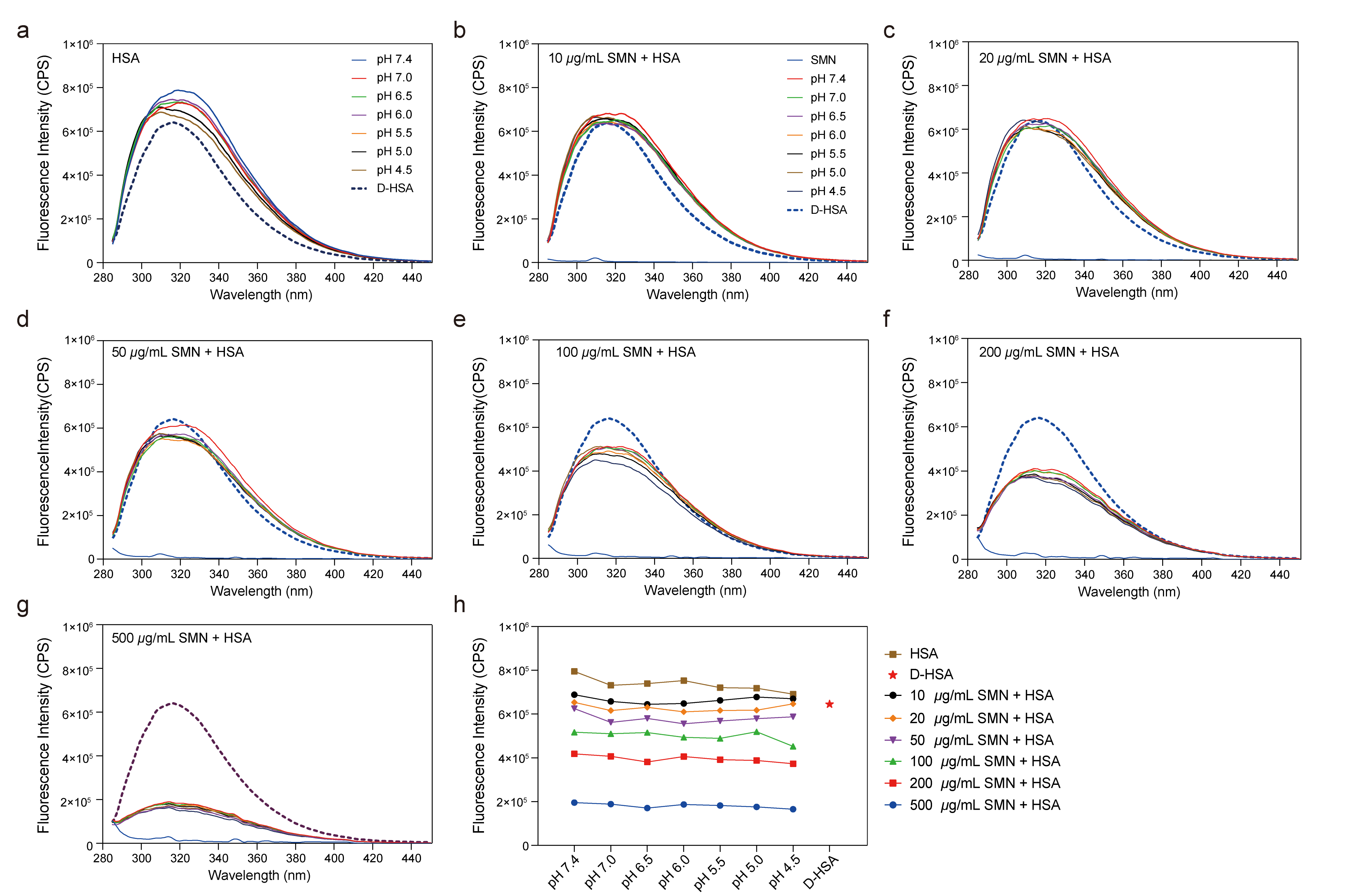
Figure S8**. **Fluorescence spectra of HSA interacting with SMNs dispersions at various concentrations**. **(a)** Fluorescence spectrum of HSA in buffer solutions. **(b-g)** Fluorescence spectra of HSA with increasing concentrations of SMNs: 10 *μ*g mL^-1^ **(b)**, 20 *μ*g mL^-1^ **(c)**, 50 *μ*g mL^-1^ **(d)**, 100 *μ*g mL^-1^ **(e)**, 200 *μ*g mL^-1^ **(f)**, and 500 *μ*g mL^-1^ **(g)** under various pH values (pH 7.4, 7.0, 6.5, 6.0, 5.5, 5.0, or 4.5). D-HSA (heat-denatured HSA, 100 °C, 10 min) is used as positive control. **(h)** Integrated plot of the maximum fluorescence intensity of HSA from spectra **(a-g).**


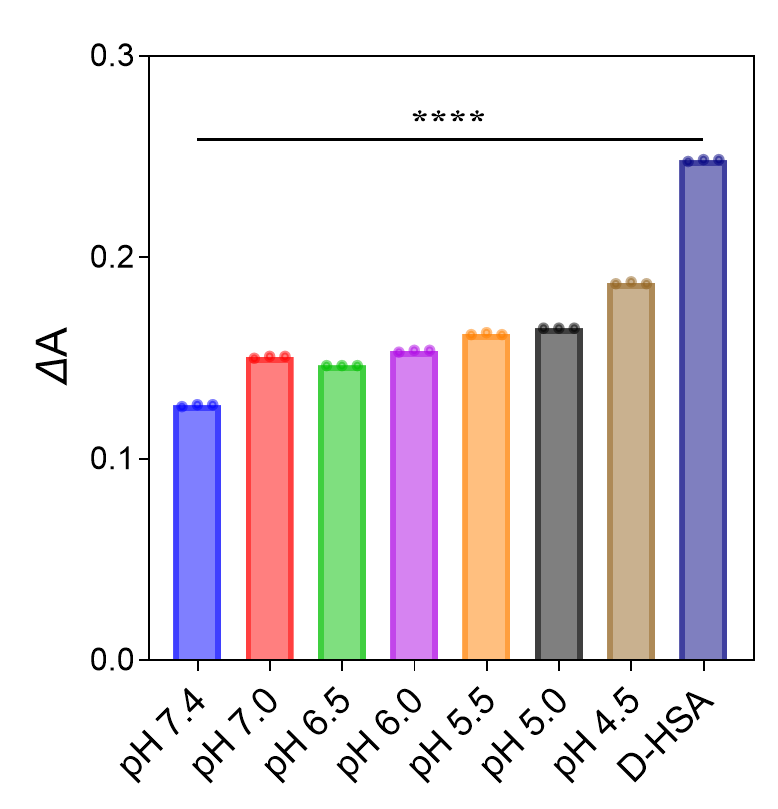


**Figure S9. Change in the maximum absorbance of HSA at 278 nm before and after adsorption of SMNs.** *Δ*A represents the difference in maximum absorbance at 278 nm before and after HSA interacts with SMNs. D-HSA (heat-denatured HSA, 100 °C, 10 min) is used as positive control. Data are presented as mean ± standard deviation with *n* = 3. Statistical significance was tested by one-way ANOVA. **p* < 0.05, ***p* < 0.01, ****p* < 0.001, *****p* <0.0001.

**
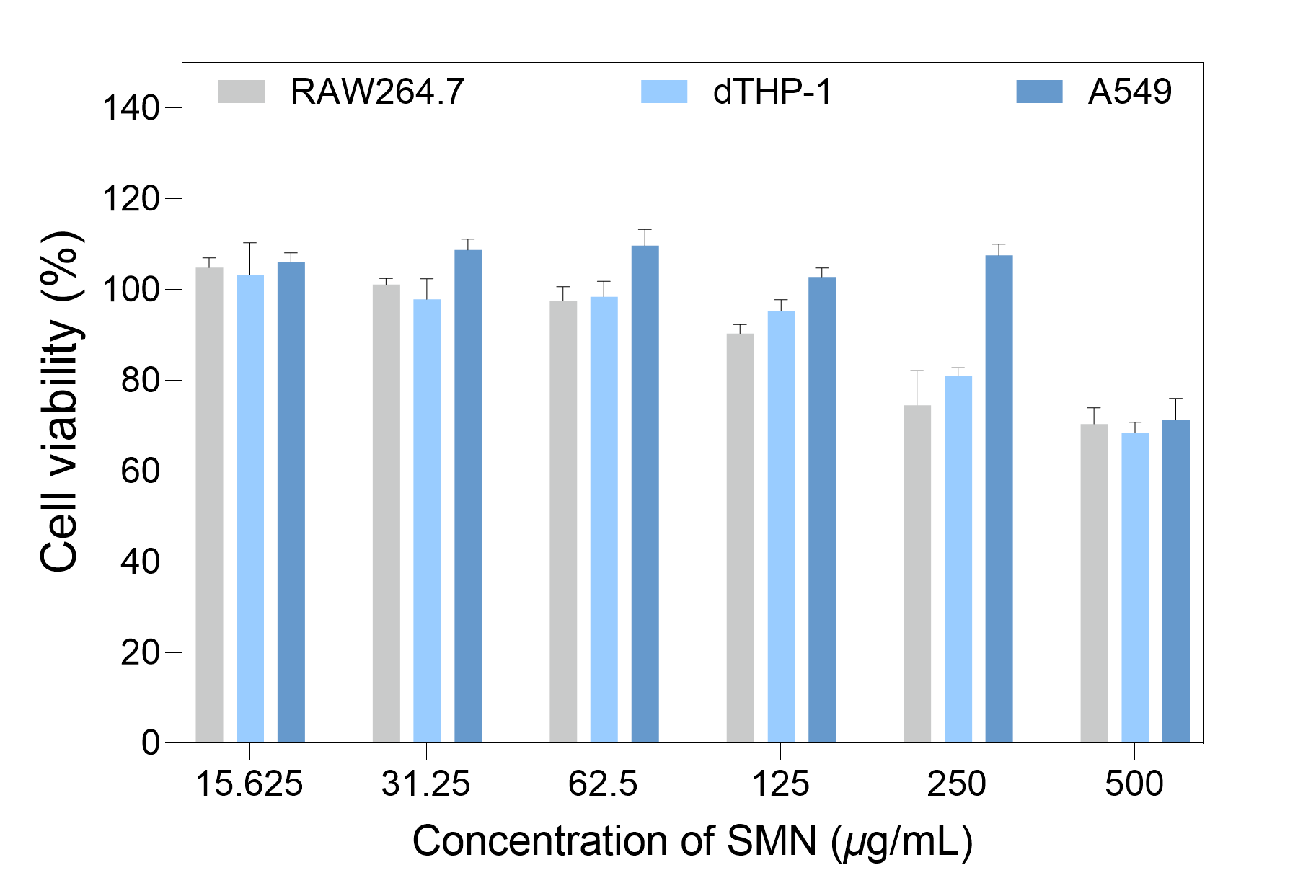
**

**Figure S10. Cell viability assessment of SMNs on RAW264.7, dTHP-1, and A549 cells.** Data are presented as mean ± SD (*n* = 3, biologically independent experiments).


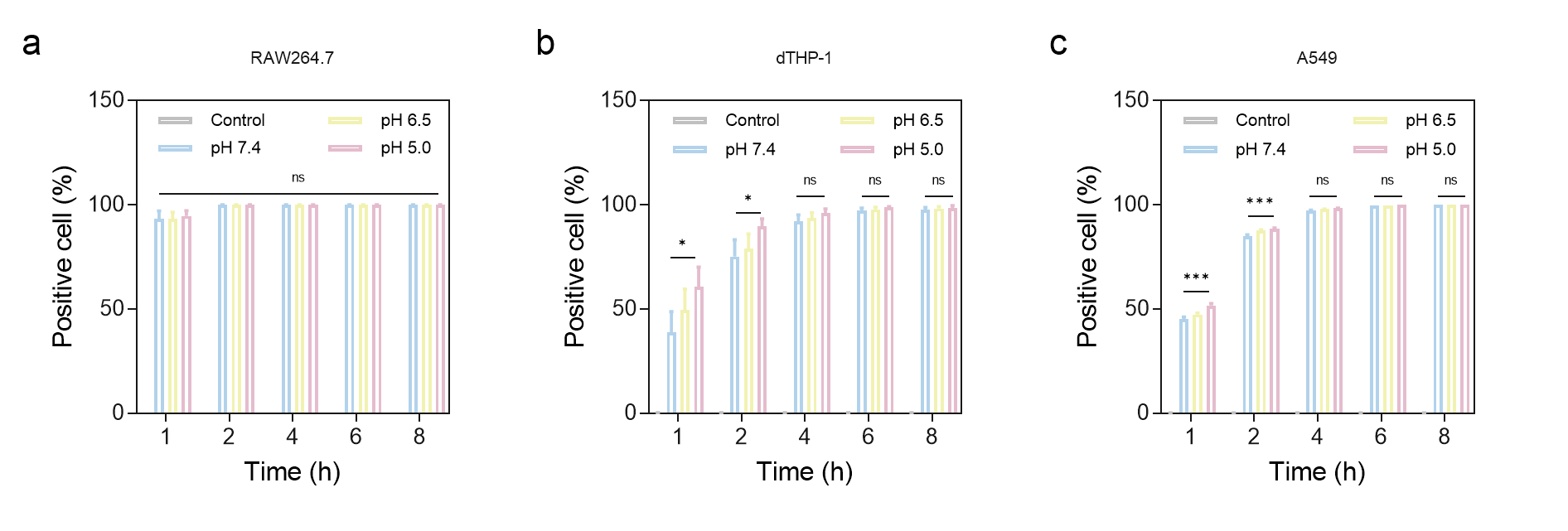
**Figure S11**. Time-dependent percentage of positive cells in RAW264.7 **(a)**, A549 **(b)**, and dTHP-1 **(c)** cells after exposure to PC@SMNs at pH 7.4, 6.5, and 5.0 for 1 h, 2 h, 4 h, 6 h, and 8 h, as determined by flow cytometry. Data are presented as mean ± SD (*n* = 3). Statistical significance was tested by one-way ANOVA. **p* < 0.05, ***p* < 0.01, ****p* < 0.001, *****p* <0.0001.

**
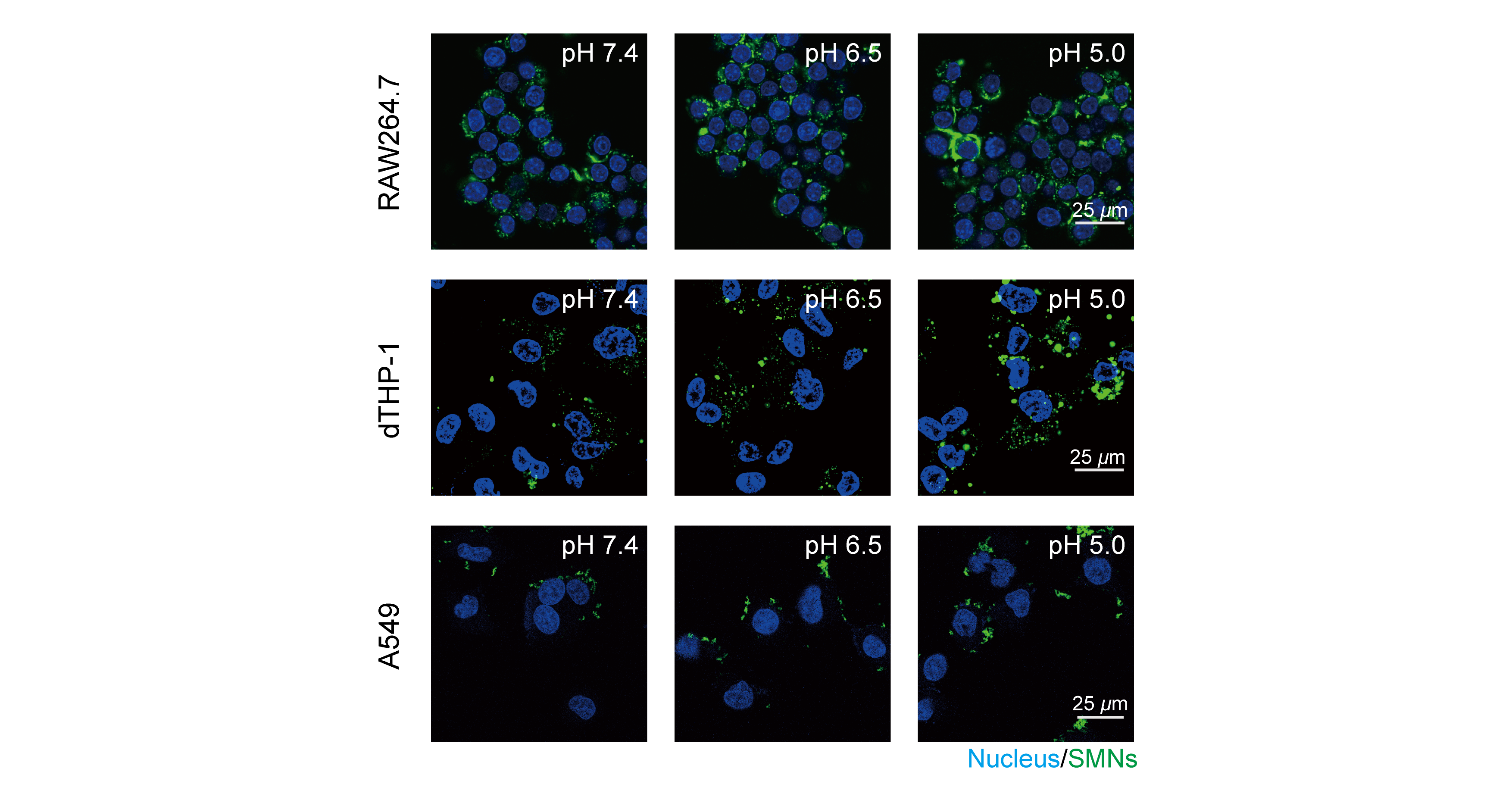
**

**Figure S12.** CLSM images showing the uptake of PC@SMNs by RAW264.7, dTHP-1, and A549 cells in serum-free medium at 2 h. SMNs were labelled with FITC (green); nuclei were stained with DAPI (blue).

**
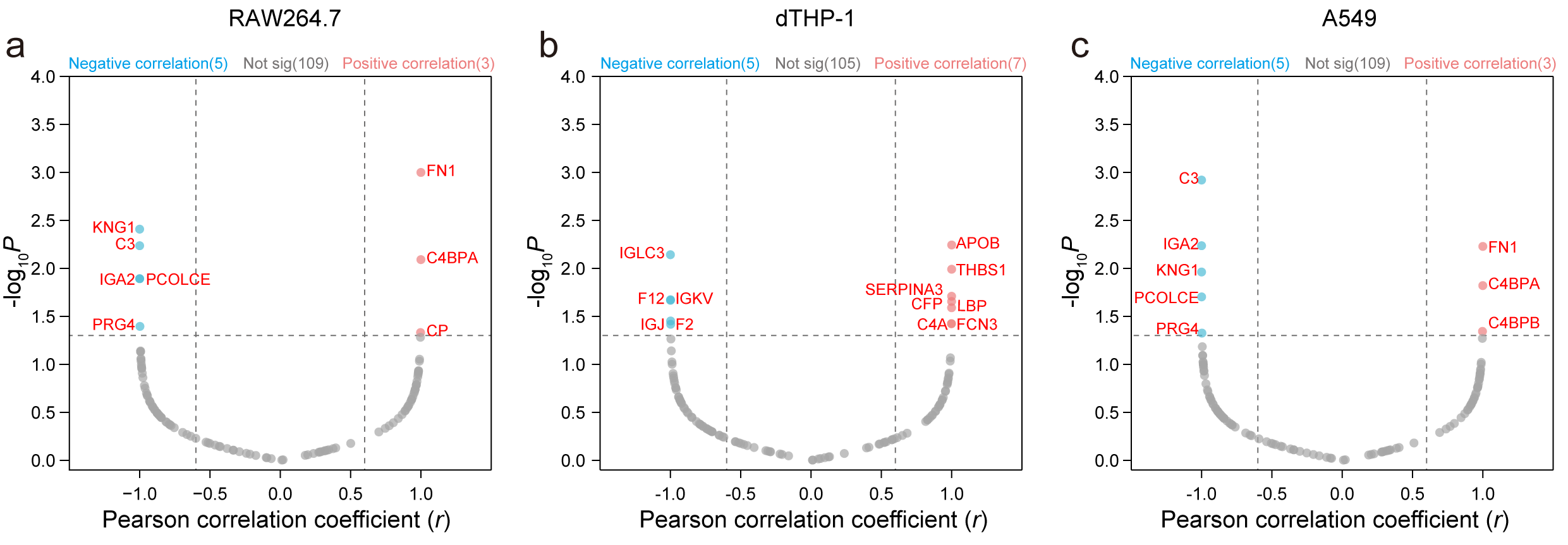
**

**Figure S13. Correlation between the cellular uptake and type of corona protein found in PC@SMNs under different pH values.** The proteins have been selected from different categories that showed large positive or negative correlation. Pearson correlation coefficients (*r*) are plotted against -log_10_*P*. The horizontal dashed line represents *P* = 0.05, and the vertical dashed lines represent *r* = 0.6 or *r* = -0.6. **(a)** RAW264.7 cells. **(b)** dTHP-1 cells. **(c)** A549 cells.

**
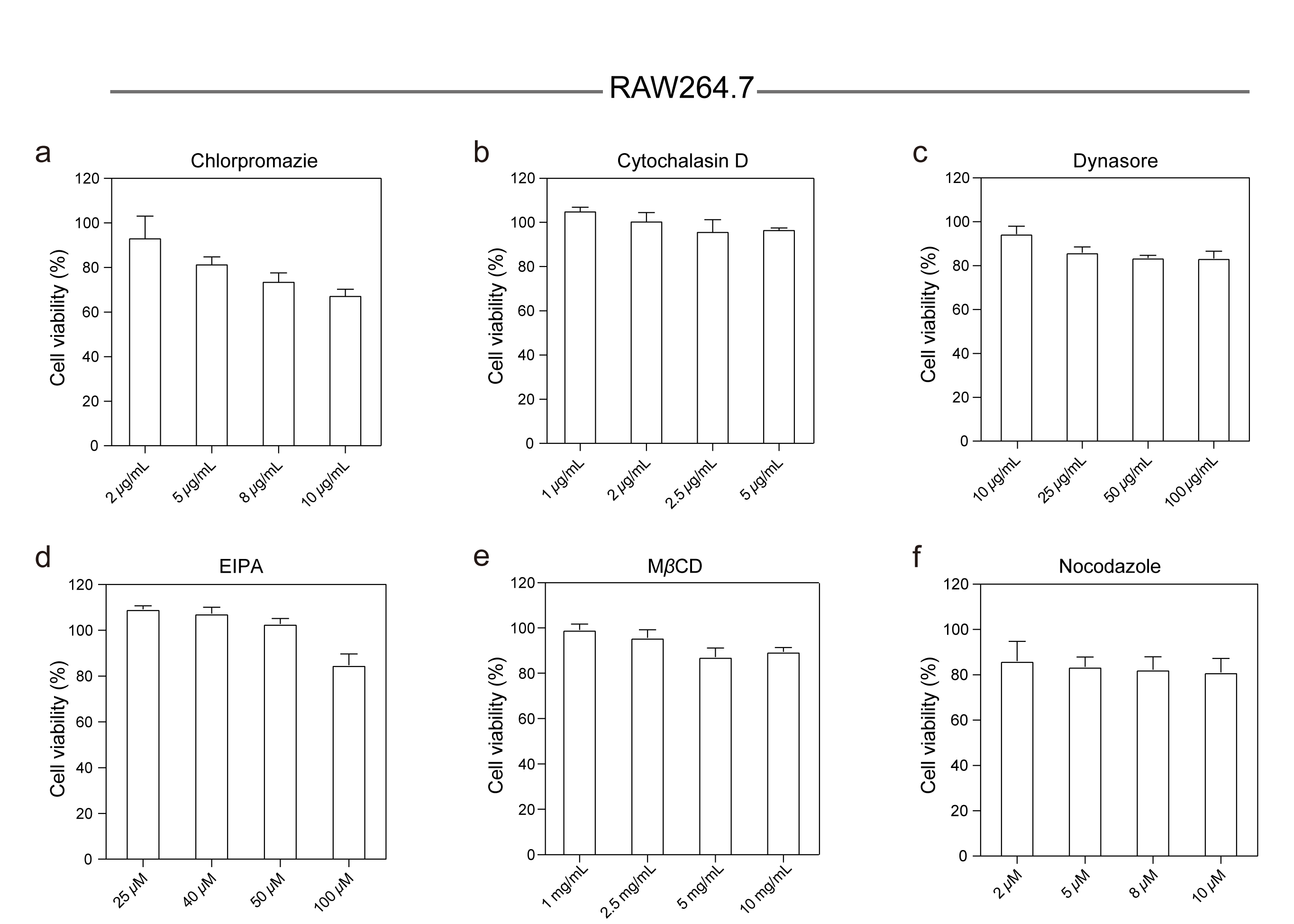
Figure S14. Cell viability of RAW264.7 cells after treatment with different concentrations of inhibitors assessed by CCK assay**. RAW264.7 cells were incubated for 1 h with chlorpromazine **(a)**, cytochalasin D **(b)**, dynasore **(c)**, EIPA **(d)**, M*β*CD **(e)**, or nocodazole **(f)** in DMEM medium. Data are normalized to control cells without inhibitors. Data are presented as mean ± SD (*n* = 3, biologically independent experiments). These results were used to determine the optimal drug concentration for each inhibitor and cell type, and exclude toxicity.

**
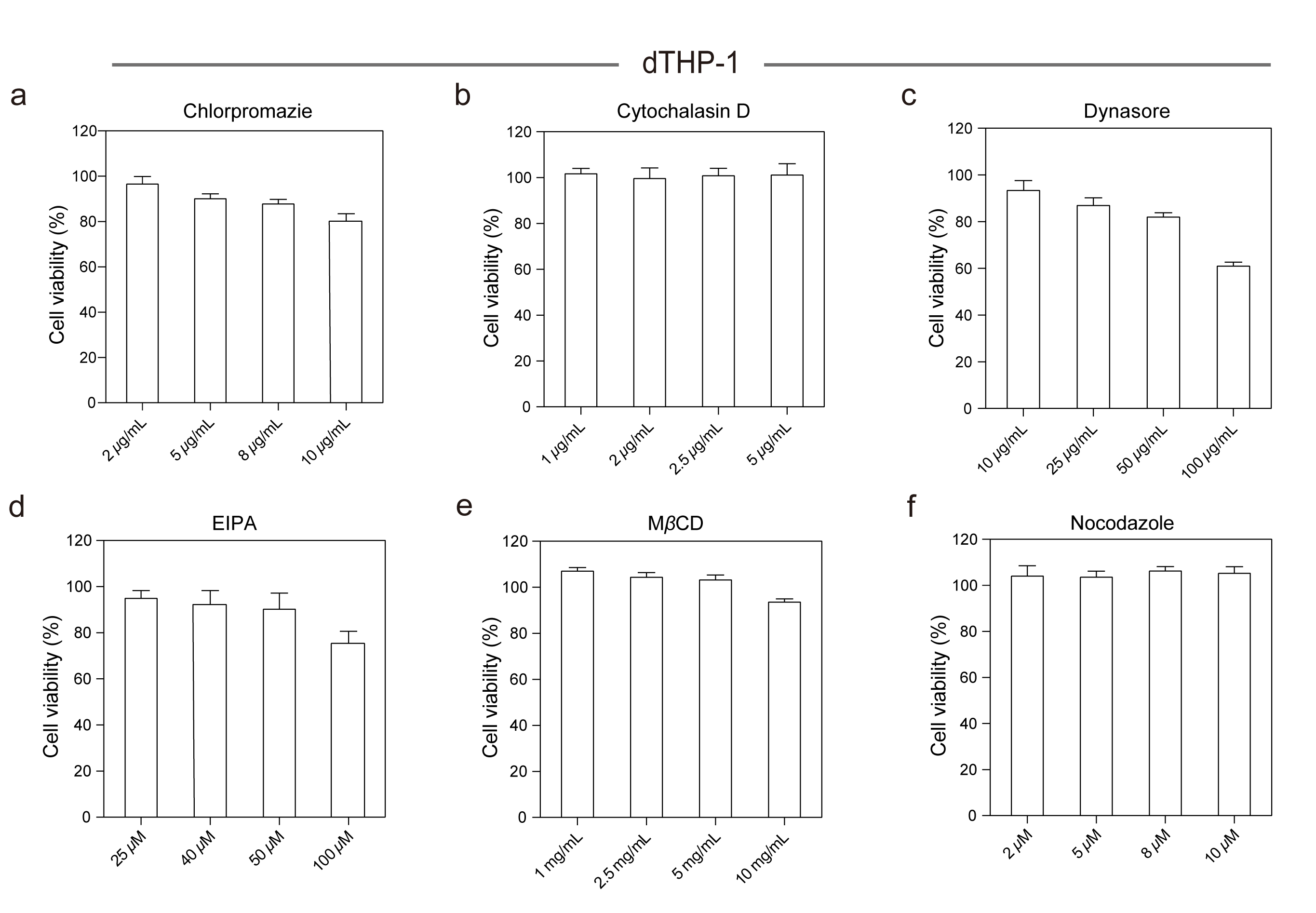
Figure S15. Cell viability of dTHP-1 cells after treatment with different concentrations of inhibitors assessed by CCK assay**. dTHP-1 cells were incubated for 1 h with chlorpromazine **(a)**, cytochalasin D **(b)**, dynasore **(c)**, EIPA **(d)**, M*β*CD **(e)**, or nocodazole **(f)** in RPMI-1640 medium. Data are normalized to control cells without inhibitors. Data are presented as mean ± SD (*n* = 3, biologically independent experiments). These results were used to determine optimal drug concentration for each inhibitor and cell type, and exclude toxicity.

**
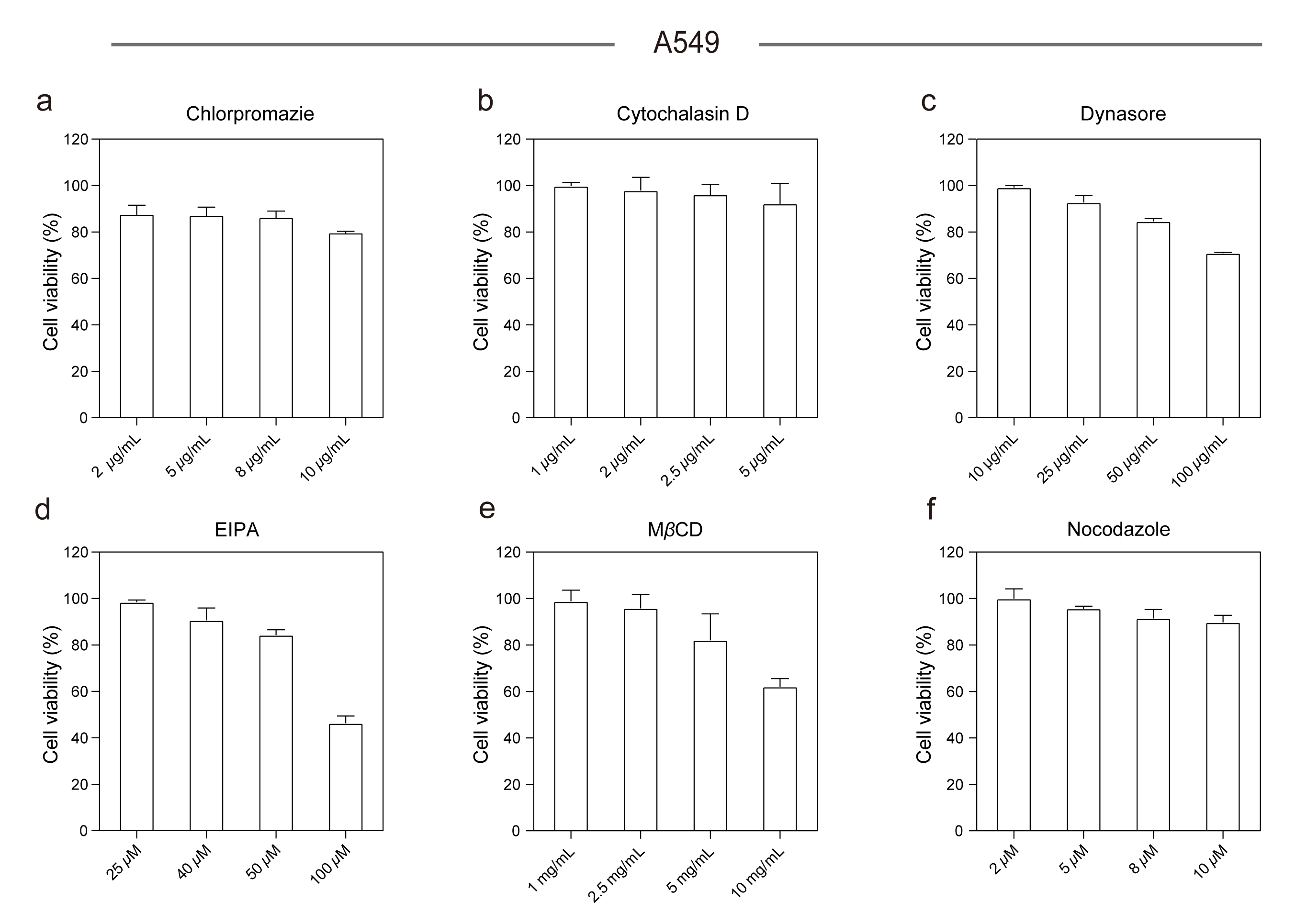
Figure S16. Cell viability of A549 cells after treatment with different concentrations of inhibitors assessed by CCK assay**. A549 cells were incubated for 1 h with chlorpromazine **(a)**, cytochalasin D **(b)**, dynasore **(c)**, EIPA **(d)**, M*β*CD **(e)**, or nocodazole **(f)** in RPMI-1640 medium. Data are normalized to control cells without inhibitors. Data are presented as mean ± SD (*n* = 3, biologically independent experiments). These results were used to determine optimal drug concentration for each inhibitor and cell type, and exclude toxicity.
